# Immune checkpoint inhibitors and cardiovascular toxicity: immunology, pathophysiology, diagnosis, and management

**DOI:** 10.1007/s11239-025-03146-7

**Published:** 2025-07-17

**Authors:** Richard C. Becker

**Affiliations:** Cardiovascular-Oncology Program, University of Cincinnati Cancer Center, Cincinnati, OH 45267 USA

**Keywords:** Immune check point inhibitors, Cardiovascular toxicity, Thrombosis, Immunology of the Heart

## Abstract

Immune checkpoint inhibitors (ICIs) are pivotal in cancer therapy, particularly but not solely for metastatic and advanced lung cancer. These monoclonal antibodies, targeting programmed cell death (PD)-1, ligand PD-L1, and cytotoxic T-lymphocyte antigen (CTLA)-4, enhance immune responses against tumors but can also trigger immune-related adverse events, including cardiotoxicity and vascular toxicity. Cardiotoxic effects, such as myocarditis, pericarditis, atrial arrhythmias, thrombosis, and vasculitis are significant concerns, particularly myocarditis that can be fatal. ICIs like pembrolizumab, nivolumab, and atezolizumab are widely used, with combination immunotherapy showing improved survival but higher myocarditis risk. Effective management of ICI-induced cardiovascular toxicity involves regular monitoring for physical findings, cardiac, inflammatory, and autoimmune biomarkers, electrocardiograms, CT angiograms, echocardiograms, and cardiac MRI as needed. Emergent treatment for ICI myocarditis and vasculitis includes immediate discontinuation of ICIs, high-dose corticosteroids, and supportive care. In severe or steroid-refractory cases, additional immunosuppressive therapies should be considered.

## Highlights

- Immune checkpoint inhibitors (ICIs) like pembrolizumab and nivolumab can cause serious cardiovascular toxicities—especially myocarditis, which is rare but often fatal and typically occurs early in treatment.
- Cardiotoxicity is driven by CD8+ T-cell and macrophage-mediated inflammation, with loss of immune tolerance and genetic predispositions (e.g., HLA-DRB1*11:01, TTN mutations) playing key roles.
- High-sensitivity troponin T (hs-cTnT) is the preferred biomarker for early detection of ICI-associated myocarditis due to its sensitivity and prognostic value.
- First-line treatment involves immediate ICI discontinuation and high-dose corticosteroids, with escalation to immunosuppressants like tocilizumab or abatacept in refractory cases.
- Next-generation ICIs aim to reduce toxicity through tumor-selective delivery systems (e.g., pH-sensitive PEGylation, microneedle patches) and bispecific antibodies to enhance precision and safety.

## Introduction

Immune checkpoints (IC) are immunosuppressive molecules that protect human tissues and organs by regulating the immune response to maintain tolerance. Cancer cells exploit these checkpoints to avoid immune recognition and destruction. Immune checkpoint inhibitors (ICIs), such as antibodies targeting programmed death (PD)-1, programmed death ligand (PDL)-1, and cytotoxic T-lymphocyte antigen (CTLA)-4, prevent these molecules from suppressing the immune system, thereby enabling the immune system to attack and kill tumor cells [1].

ICIs are widely used in the treatment of non-small cell lung cancer, particularly for metastatic and advanced stage disease, melanoma, hepatocellular cancer, gastric cancer, head and neck cancer, and Hodgkin’s lymphoma [2]. Currently, ICIs are approved for use in 20 different types of cancer (reviewed in Alturki NA) [3]. However, an immune response to heart-related antigens and several other mechanisms can lead to serious cardiotoxicity [4], including myocarditis, pericardial disease, non-inflammatory left ventricular dysfunction [5], myocardial infarction (MI) [6], arrhythmias, left ventricular mural thrombosis, thromboembolism, and vasculitis with or without in situ thrombosis.

The purpose of this targeted review is to summarize the current ICIs and focus on their immunological properties, distinction between first- and second-generation drugs, pathophysiology of their cardiovascular toxic effects, suggested screening and surveillance strategies, and optimal management in clinical practice.

## A foundation for immunology and immunopathology in cancer

*Systemic* and *tissue-specific* immunology in humans is built on the capacity of the immune system to distinguish ‘self from non-self’, deploying both innate and adaptive mechanisms. Systemic immunity involves circulating immune cells and soluble mediators that provide broad surveillance and rapid responses, while tissue-specific immunity is shaped by local antigen-presenting cells, tissue-resident lymphocytes, and the unique microenvironmental cues of each organ. Evolutionarily, these layered immune systems have developed to optimize host defense against pathogens while minimizing collateral tissue damage, with immune checkpoints such as CTLA-4 and PD-1 evolving as critical regulators to prevent autoimmunity by restraining T-cell activation and maintaining self-tolerance [7].

The *thymus* is the central organ for T cell development, where bone marrow-derived progenitors undergo selection processes that generate a diverse, self-tolerant T cell repertoire essential for both systemic and tissue-specific immune responses. Through positive and negative selection, the thymus ensures that emerging T cells can recognize foreign antigens presented by self-MHC while eliminating or diverting those with high affinity for self-antigens, thus establishing central tolerance and preventing autoimmunity. The thymic microenvironment, composed of specialized epithelial and stromal cells, orchestrates the differentiation of conventional T cells, regulatory T cells, and minor subsets that contribute to immune homeostasis across tissues [8], [9].

In the context of cancer, thymic involution, whether age-related or therapy-induced, leads to reduced output of naïve T cells, a restricted TCR repertoire, and a relative increase in regulatory T cells, collectively impairing immune surveillance and facilitating tumor immune evasion. This dysregulation increases susceptibility to malignancy and can alter the balance between effective anti-tumor immunity and tolerance, contributing to both cancer progression and immune escape. Tthe thymus is highly conserved as it enables the adaptive immune system to balance diversity and self-tolerance, a trade-off that underpins both robust pathogen defense and the risk of immune dysregulation in cancer and autoimmunity [10, 11].

Cancer induces *immune dysregulation* through a combination of local and systemic mechanisms that lessen both innate and adaptive immunity. At the tissue level, malignant cells alter the tumor microenvironment (TME) by recruiting immunosuppressive cell populations such as regulatory T cells (Tregs), myeloid-derived suppressor cells (MDSCs), and M2-polarized macrophages, while simultaneously downregulating antigen presentation machinery and secreting immunosuppressive cytokines (e.g., TGF-β, IL-10). These changes create an environment that impairs cytotoxic T cell and natural killer (NK) cell function, promotes immune tolerance, and facilitates tumor progression. Tumor cells also exploit metabolic competition, hypoxia, and extracellular vesicle release to further suppress local immune responses and spread immunosuppressive signals systemically [12, 13].

Systemically, cancer-associated inflammation leads to chronic production of pro-inflammatory cytokines and acute phase reactants, which can drive paraneoplastic syndromes and alter immune cell development and trafficking [14]. Tumor-derived factors can induce myelopoiesis, skewing hematopoiesis toward immunosuppressive phenotypes, and disrupt the balance between effector and regulatory immune cells throughout the body. This systemic immune dysregulation is reinforced by the evolutionary process of immunoediting, in which immune pressure selects for tumor clones with reduced immunogenicity or enhanced immune evasion capabilities, resulting in increased intra-tumoral heterogeneity and resistance to immune-mediated elimination [15].

From an evolutionary biology perspective, these mechanisms reflect the dynamic co-evolution of host immune surveillance and tumor escape strategies. The immune system initially eliminates highly immunogenic tumor cells, but persistent immune activation selects for variants that are non-identifiable to the immune system or develop cytoprotective adaptations, ultimately allowing immune escape and tumor outgrowth [16].

### Immune system of the heart

The heart has its own immune system, comprising various immune cells that play crucial roles in maintaining cardiac homeostasis and responding to injury [17]. These immune cells are involved in essential housekeeping functions, such as removing dying tissue, scavenging pathogens, and promoting healing after myocardial infarction or infection. The heart contains a diverse array of immune cells, including macrophages, dendritic cells, T cells, and B cells, which contribute to both the defense against pathogens and the maintenance of normal cardiac function [18–20].

**Fig. 1:**
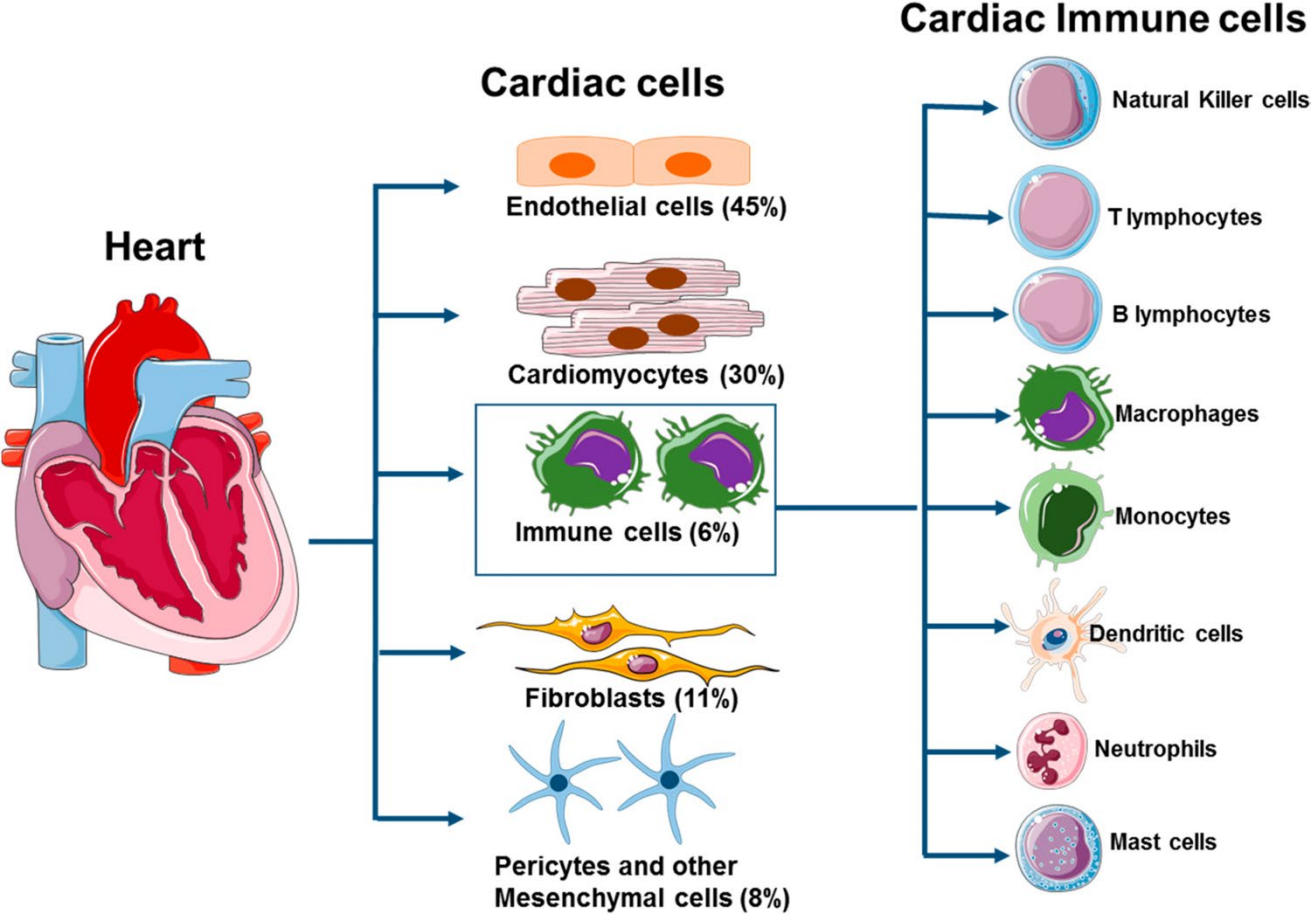
Immune System of the Heart. The heart has its own immune system, comprising various immune cells that play crucial roles in maintaining cardiac homeostasis and responding to injury. These immune cells are involved in essential housekeeping functions, such as removing dying tissue, scavenging pathogens, and promoting healing after myocardial infarction or infection. The primary immune cells include macrophages, dendritic cells, T cells, and B cells. Immune cells from the peripheral blood can also migrate to the heart when needed. The principal innate immune cells in the heart are *resident macrophages*, which perform phagocytosis of pathogens and apoptotic cells, regulate inflammation, and contribute to tissue repair. *Dendritic cells* are present in the myocardium and function as antigen-presenting cells, bridging innate and adaptive immunity by activating T cells. *Neutrophils* and *mast cells* are rapidly recruited during acute injury or infection, contributing to pathogen clearance and modulation of inflammation, but can also exacerbate tissue damage if uncontrolled. The adaptive immune system in the heart is primarily represented by *T lymphocytes* (including Th1, Th17, and regulatory T cells) and *B lymphocytes*. T cells orchestrate immune responses against infected or malignant cells, with regulatory T cells (Tregs) limiting excessive inflammation and promoting repair. B cells contribute to antibody-mediated immunity and modulate inflammation. *Natural killer (NK) cells* provide surveillance against virally infected and transformed (cancerous) cells, contributing to anti-tumor immunity within the cardiac microenvironment. See text. From: Lafuse, W.P.; Wozniak, D.J.; Rajaram, M.V.S. Role of Cardiac Macrophages on Cardiac Inflammation, Fibrosis and Tissue Repair. *Cells* 2021, *10*, 51. 10.3390/cells10010051

The heart is considered an immune-privileged organ with defenses that attenuate baseline and stimulated immune responses [21]. Relatively few T cells are present under basal conditions. In addition, T cell–mediated injury to the heart is reduced by negative-feedback loops that operate in the myocardium. These feedback loops include the secretion of the cytokine IFN-γ from Th1 cells and CTLs, resulting in PD-L1 upregulation on cardiac endothelial cells; PD-L1 then suppresses effector T cells [22]. Immune cells found within the heart and their biological functions are summarized in [23] (Fig. 1).

### Location, migration and activation

Immune cells in the heart are found in various regions, each contributing to cardiac homeostasis and response to injury. Specific locations include the myocardium, which is the primary site where macrophages, T cells, and dendritic cells are found [24]. In the pericardium, B cells and macrophages are commonly found, contributing to immunosurveillance and inflammatory responses [25]. The coronary endothelium contains macrophages, mast cells, and dendritic cells, which play roles in vascular health and immune responses. Specific populations of macrophages are also found in the valves and AV node [26], where they are involved in maintaining the structural integrity and function of these areas [27].

Immune cells from the peripheral blood can migrate to the heart, particularly during cardiac injury or infection [28]. This migration is essential for amplifying the immune response. For instance, after a myocardial infarction, monocytes from the bloodstream migrate to the heart and differentiate into macrophages to aid in tissue repair [29]. These migrating immune cells can either be already activated or become activated upon reaching the heart. The activation process involves recognizing damage-associated molecular patterns (DAMPs) or pathogen-associated molecular patterns (PAMPs), which then trigger an immune response [30] [31].

### Immunity of the vascular system

Studies have shown that the PD-1/PD-L1 pathway plays a significant role in maintaining vascular immunity and regulating T cell activation within the vascular endothelium. Endothelial cells express PD-L1, which interacts with PD-1 on T cells to inhibit their activation and cytolytic activity. This interaction helps to prevent excessive immune responses that could damage the vascular endothelium [32].

## Immune checkpoint inhibitors

### How do immune checkpoint inhibitors work against cancer?

As summarized previously, immune checkpoints are a normal part of human immunity. Their role is to prevent an immune response from being so strong that it destroys healthy cells. Immune checkpoints engage when proteins on the surface of T cells recognize and bind to partner proteins on other cells, such as tumor cells. These proteins are called IC proteins. When the checkpoint and partner proteins bind together, they trigger an “off” signal to the T cells. This can prevent the immune system from destroying cancer cells. This is referred to as tumor cell escape.

Immune checkpoint inhibitors work by blocking IC proteins from binding with their partner proteins, preventing the “off” signal from being sent, and enabling T cells to eradicate cancer cells [33–35].

### Drug development: a brief history

The first step in the development of ICIs for cancer therapy was the identification of specific antigens that an antibody would target. Common targets include PD-1, PDL-1, and CTLA-4. These antigens are selected based on their role in immune suppression and their expression on cancer cells. The development of drugs for use in patient care involved many foundational stages [36, 37].

The synthesis of ICIs involves a combination of traditional and contemporary biotechnological methods. From antigen identification to purification and testing, each step is crucial in producing effective and safe monoclonal antibodies for cancer treatment [38].

First-generation ICIs include anti-CTLA-4 (ipilimumab) and anti-PD-1/PD-L1 agents (nivolumab, pembrolizumab, atezolizumab, avelumab, durvalumab, cemiplimab). Second-generation ICIs target additional checkpoints such as LAG-3 (relatlimab), with others (e.g., TIM-3, TIGIT) in development. These agents differ in their immunological effects and toxicity profiles (Table 1).

**Table 1:** Immune checkpoint inhibitors

Generation	Drug (Target)	Unique properties	Immunological response	Cardiovascular toxicity (frequency, onset)	Uptake in clinical practice	Most common cancers treated
First-generation	Ipilimumab (CTLA-4)	Blocks CTLA-4; early T-cell priming	Broad T-cell activation	Myocarditis, pericarditis, arrhythmia (2–3%, < 3 mo)	High	Melanoma, RCC, CRC, HCC
First-generation	Nivolumab (PD-1)	Blocks PD-1; peripheral action	Restores T-cell function in TME	Myocarditis, pericarditis, arrhythmia (0.5–1%, < 3 mo)	High	Melanoma, NSCLC, RCC, HCC, HL, HNSCC, UC
First-generation	Pembrolizumab (PD-1)	Blocks PD-1; like nivolumab	Restores T-cell function in TME	Myocarditis, pericarditis, arrhythmia (0.5–1%, < 3 mo)	High	Melanoma, NSCLC, HL, HNSCC, UC, GC, MSI-H tumors
First-generation	Cemiplimab (PD-1)	Blocks PD-1; newer PD-1 agent	Like other PD-1 inhibitors	Myocarditis, pericarditis (rare, < 3 mo)	Moderate	CSCC, NSCLC
First-generation	Atezolizumab (PD-L1)	Blocks PD-L1; prevents PD-1/PD-L1 interaction	Restores T-cell function in TME	Myocarditis, pericarditis (rare, < 3 mo)	Moderate	NSCLC, UC
First-generation	Avelumab (PD-L1)	Blocks PD-L1; like atezolizumab	Restores T-cell function in TME	Myocarditis, pericarditis (rare, < 3 mo)	Low-Moderate	Merkel cell carcinoma, UC
First-generation	Durvalumab (PD-L1)	Blocks PD-L1; like atezolizumab	Restores T-cell function in TME	Myocarditis, pericarditis (rare, < 3 mo)	Moderate	UC, NSCLC
Second-generation	Relatlimab (LAG-3)	Blocks LAG-3; T-cell exhaustion modulator	Enhances T-cell activation	Myocarditis possible (rare, unknown onset)	Low (emerging)	Melanoma (in combination)
First/Second-gen Combo	Ipilimumab + Nivolumab	Dual checkpoint blockade	Synergistic T-cell activation	Myocarditis, pericarditis, arrhythmia (up to 3–5%, < 3 mo)	Moderate	Melanoma, RCC, others

*TLA-4* Cytotoxic T-Lymphocyte Antigen 4; *PD-1* Programmed Death-1; *PD-L1* Programmed Death-Ligand 1; *LAG-3* Lymphocyte Activation Gene-3; *TME* Tumor Microenvironment; *RCC* Renal Cell Carcinoma; *CRC* Colorectal Cancer; *HCC* Hepatocellular Carcinoma; *NSCLC* Non-Small Cell Lung Cancer; *HL* Hodgkin Lymphoma; *HNSCC* Head and Neck Squamous Cell Carcinoma; *UC* Urothelial Carcinoma; *GC* Gastric Cancer; *MSI-H* Microsatellite Instability-High; *CSCC* Cutaneous Squamous Cell Carcinoma

### Clinical indications

Immune checkpoint inhibitors (ICIs) have become a cornerstone in cancer therapy, with a significant number of patients in the United States (US), Europe, and Asia receiving these treatments. According to recent estimates, approximately 43.6% of US cancer patients are eligible for ICIs representing more than 20 different cancers [39]. The most common cancers treated include melanoma, non-small cell lung cancer (NSCLC), renal cell carcinoma, head and neck squamous cell carcinoma, and bladder cancer [40].

### Common ICI combinations

Immune checkpoint inhibitors (ICIs) are used in combination with chemotherapy and radiation therapy for cancer treatment, including for non-small cell cancer, head and neck squamous cell cancer, cervical cancer, and esophageal cancer. Toxicity resulting from the combined use of chemotherapy, radiation, and ICIs must always be considered in clinical practice [41].

Immune checkpoint inhibitors (ICIs) can also be used in combination. Common ICI combinations include nivolumab (PD-1 inhibitor) and ipilimumab (CTLA-4 inhibitor), used for melanoma, renal cell carcinoma, and non-small cell lung cancer. Another combination is pembrolizumab (PD-1 inhibitor) and axitinib (VEGF-vascular endothelial growth factor inhibitor), used for renal cell carcinoma, leveraging the anti-angiogenic effects of axitinib to enhance anti-tumor immune response. Atezolizumab (PDL-1 inhibitor) combined with bevacizumab (VEGF inhibitor) is used for the treatment of hepatocellular carcinoma. Lastly, durvalumab (PD-L1 inhibitor) and tremelimumab (CTLA-4 inhibitor) are used for non-small cell lung cancer and hepatocellular carcinoma, enhancing the immune response by targeting different checkpoints [42–44].

Combination regimens, especially CTLA-4 plus PD-1/PD-L1, confer the highest risk of severe cardiovascular immune related adverse events (irAEs), with myocarditis being the most frequent and often presenting early in the treatment course. Vigilance for early symptoms and prompt management are critical, as recommended by the American Society of Clinical Oncology [45].

## Immune checkpoint inhibitor toxicity

### General constructs

When ICIs block proteins like programmed death (PD)-1, programmed death ligand (PDL)-1, and cytotoxic T-lymphocyte antigen (CTLA)-4, they remove the “brakes” on T cells, allowing them to become more active. In some cases, other immune cells like B cells and macrophages can also be involved in the immune response. As previously summarized, the primary target of the augmented immune response is cancer cells, with the goal of ICIs being to enhance the immune system’s ability to recognize and destroy these cells. Immune activation is not tumor-specific, and as a result, most patients will experience some form of adverse reaction. Most immune responsive adverse events (irAEs) involve the skin and gastrointestinal (GI) tract; however, any organ can be involved that include inflammation of the heart (myocarditis and pericarditis), conduction block and arrhythmias, atrial myopathy, arterial and venous thrombosis, blood vessels (vasculitis), lungs (pneumonitis), liver (hepatitis), adrenal gland (adrenal insufficiency) and other organs [46–48] (Table 1).

While the focus of this review is on the cardiovascular system, clinicians must be aware of the broad range of non-cardiovascular toxicities that can directly or indirectly impact the initial presentation, clinical profile, and management of patients with multi-organ ICI toxicity (Table 2).

**Table 2:** Cardiovascular and non-cardiovascular toxicities of immune checkpoint inhibitors

Toxicity type	Time to onset	Time to resolution	Common clinical findings	Common laboratory findings	Morbidity	Mortality
*Cardiotoxicities*						
Myocarditis	Within weeks to months of starting ICIs	Variable, can be weeks to months after stopping ICIs	Chest pain, shortness of breath, fatigue	Elevated troponin, abnormal ECG, cardiac MRI findings	High	Up to 50%
Pericarditis	Weeks to months	Weeks to months	Chest pain, pericardial effusion	Elevated inflammatory markers, pericardial fluid analysis	Moderate	Low
Heart Failure	Months	Variable	Dyspnea, edema, fatigue	Elevated BNP/NT-proBNP, echocardiogram abnormalities	High	Moderate
Arrhythmias	Weeks to months	Variable	Palpitations, dizziness, syncope	Abnormal ECG, Holter monitor findings	Moderate	Low
Vasculitis (small, medium, and large vessel	Weeks to months	Variable	Fever, weight loss, fatigue, limb claudication, abdominal pain, skin ulcers, palpable purpura, aneurysm, dissection, occlusion (MI or stroke), claudication, ischemic bowel, renal failure	Elevated inflammatory markers, imaging findings (e.g., PET/CT), angiography, biopsy	Moderate	Low
*Non-Cardiotoxicities*						
Pneumonitis	Weeks to months	Weeks to months	Cough, dyspnea, fever	Ground-glass opacities on CT, elevated inflammatory markers	High	Moderate
Colitis	Weeks to months	Weeks to months	Diarrhea, abdominal pain, blood in stool	Elevated inflammatory markers, colonoscopy findings	High	Low
Hepatitis	Weeks to months	Weeks to months	Jaundice, fatigue, abdominal pain	Elevated liver enzymes, abnormal liver biopsy	Moderate	Low
Endocrinopathies (e.g., thyroiditis)	Weeks to months	Variable	Fatigue, weight changes, temperature intolerance	Abnormal thyroid function tests	Moderate	Low
Adrenal Insufficiency	Months (median 5 months)	Variable	Fatigue, weakness, anorexia, hypotension, shock	Low serum cortisol, elevated ACTH (primary AI)	Moderate	Moderate*

Brain Natriuretic Polypeptide (BNP); N-Terminal (NT); ECG (electrocardiogram); Positron Emission Tomography (PET); Computerized Tomography (CT); Magnetic Resonance Imaging (MRI); Adrenocorticotropic hormone (ACTH). * Adrenal or Addisonian crisis

### Why is the heart a target for ICI toxicity?

The heart is a site of toxicity for ICIs due to both tissue-specific and systemic inflammatory mechanisms. These fundamental constructs were summarized previously. ICIs, such as those targeting PD-1, PD-L1, and CTLA-4, can lead to irAEs including myocarditis, which is characterized by lymphocytic infiltration and myocardial inflammation [49].

Tissue-specific mechanisms involve the direct activation of T cells against cardiac antigens. For instance, cytotoxic CD8 + T cells, particularly the TEMRA subset (TEMRA cells are terminally differentiated effector T cells that are associated with protracted antigen exposure) have been implicated in ICI-induced myocarditis due to their clonal expansion and cytotoxic activity within the myocardium [49].Macrophages also play a significant role, with an expansion of inflammatory CCR2 + macrophages observed in myocarditis, driven by interactions with CD8 + T cells and IFN-γ signaling [50]. An increase in preexisting autoantibodies can contribute to autoimmune responses with toxic effects (reviewed in Postow et al.) [51].

**Fig. 2:**
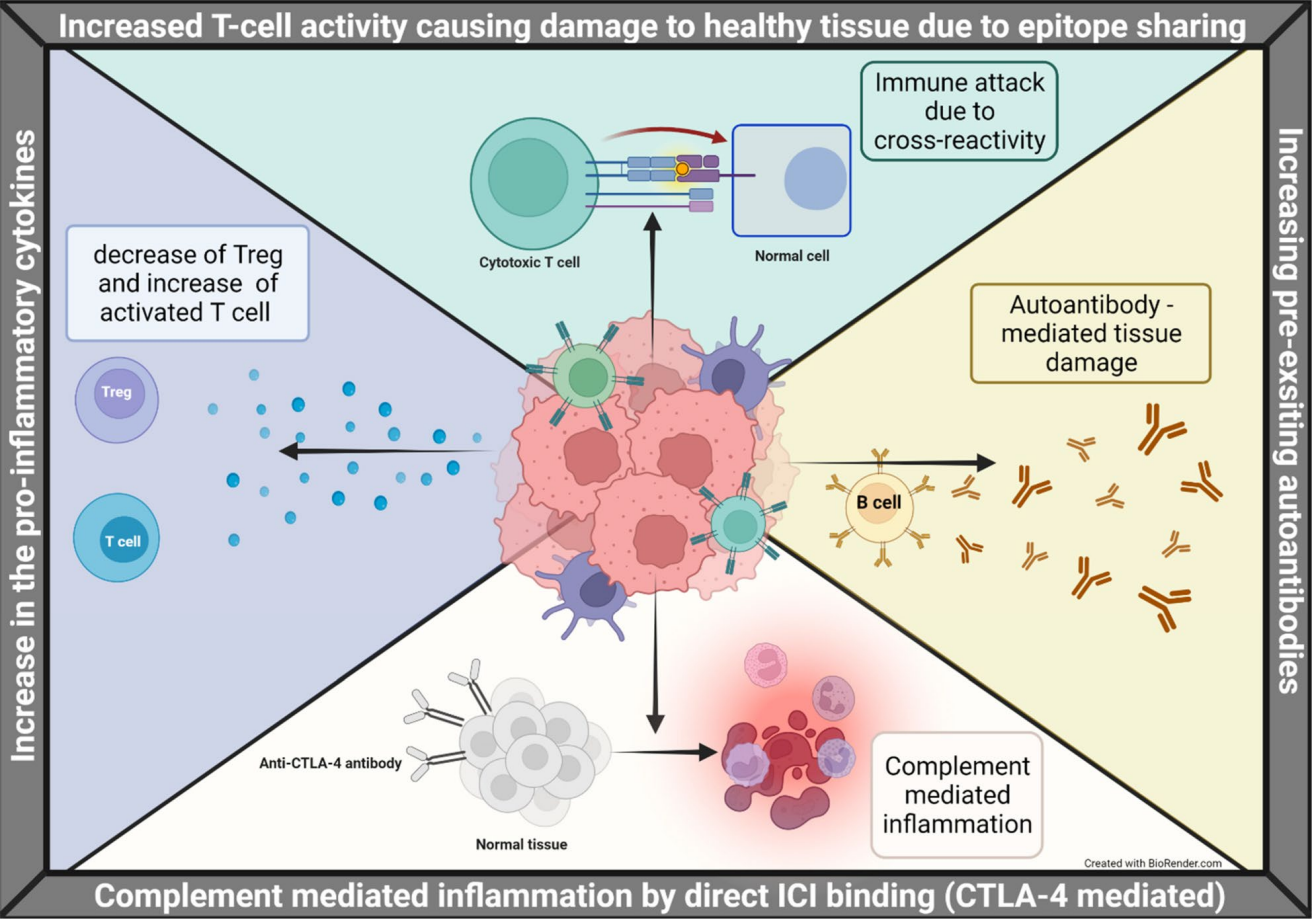
Proposed Mechanisms of Immune Checkpoint Inhibitor Toxicity. Immune checkpoint inhibitors (ICIs) and their respective ligands are shown in the context of the tumor immune microenvironment (TME). Various immune checkpoint target-mediated interactions between immune cells such as dendritic cells (DC) (serving as antigen presenting cells [APCs]), T cells, NK cells, and tumor cells are shown. ICIs disrupt immune homeostasis, leading to increased production of pro-inflammatory cytokines such as TNF-α, IL-1β, and IL-6, which can augment cardiac inflammation and dysfunction. Direct binding of antibodies against CTLA-4 with CTLA-4 expressed on normal tissues can enhance complement-mediated inflammation. From: Li, H.; Sahu, K.K.; Maughan, B.L. Mechanism and Management of Checkpoint Inhibitor-Related Toxicities in Genitourinary Cancers. Cancers 2022, 14, 2460. 10.3390/cancers14102460

Beyond tissue- based effects, systemic inflammation also contributes to cardiotoxicity. ICIs disrupt immune homeostasis, leading to increased production of pro-inflammatory cytokines such as TNF-α, IL-1β, and IL-6, which can augment cardiac inflammation and dysfunction (reviewed in Gergely et al.) [52]. Direct binding of antibodies against CTLA-4 with CTLA-4 expressed on normal tissues, such as the pituitary gland, can enhance complement-mediated inflammation (reviewed in Ronen et al.) [53, 54] (Fig. 2).

### Proposed pathogenesis of cardiotoxicity

Mechanistic studies of ICI-myocarditis have provided a greater appreciation of central and peripheral tolerance in cardiac autoimmunity. The current evidence presented above supports a model in which potential autoreactive cardiac T cells may emerge due to a lack of expression of specific cardiac antigens in thymic epithelial cells, impairing the mechanisms of central tolerance. Peripheral tolerance, via ICs, blunts priming of this group of T cells at baseline. However, the loss of this protective mechanism occurs with the use of ICIs [55, 56].

Immune checkpoint inhibitors (ICIs) facilitate the priming of autoreactive T cells through the interaction of CD28 on the T cell surface with CD80 and CD86 on professional antigen-presenting cells (APCs) in lymphoid tissue. These primed T cells then circulate in the periphery and, upon encountering cognate cardiac antigens via TCR–MHC interactions, become activated to display effector functions based on their subset. Autoreactive cardiac T cells may undergo clonal expansion, significantly increasing the proportion of T cells targeting cardiac antigens and driving myocardial injury. In response to cardiac inflammation and injury, cardiomyocytes upregulate cell surface PD-L1. However, blockade via anti-PD1 or anti-PD-L1 therapy disrupts appropriate IC signaling, potentially exacerbating cardiac injury [57–59].

The interaction between the heart's immune system (summarized below) and ICI toxicity involves the expansion of pathogenic cardiac macrophages, particularly CCR2 + monocyte-derived macrophages, which release pro-inflammatory chemokines like CXCL9 and CXCL10. These macrophages interact with activated CD8 + T-cells via IFN-γ signaling, exacerbating myocardial inflammation and damage [50].

In addition, ICIs impair the efferocytosis function of macrophages by inducing the cleavage of MerTK, a receptor that is crucial for phagocytic regulation, leading to sustained inflammation and impaired tissue repair [60]. The cGAS-STING pathway is also activated in macrophages, promoting their polarization into a pro-inflammatory phenotype, further contributing to myocarditis [61]. The immune system of the heart represents a foundation for dysregulated responses and toxic effects.

The evidence for cardiac toxicity of ICIs is based on human case reports and case series, pathological specimens (including myocardial biopsies), pharmacovigilance data, and meta-analyses of randomized controlled trials [62]. Animal models and mechanistic studies have provided additional insights into pathogenesis [63], but clinical recognition and regulatory action are grounded in human data.

The US Food and Drug Administration (FDA) and European Medicine Agency (EMA) have relied on human case reports, post-marketing surveillance, and clinical trial safety data for package inserts and approvals regarding ICI-associated cardiac toxicity. These agencies do not base regulatory decisions on in silico models or animal studies alone, but rather on observed human adverse events and supporting pathological evidence [64].

## Is the mechanism of cardiotoxicity unique to the heart compared with other tissues and organ systems?

**Fig. 3:**
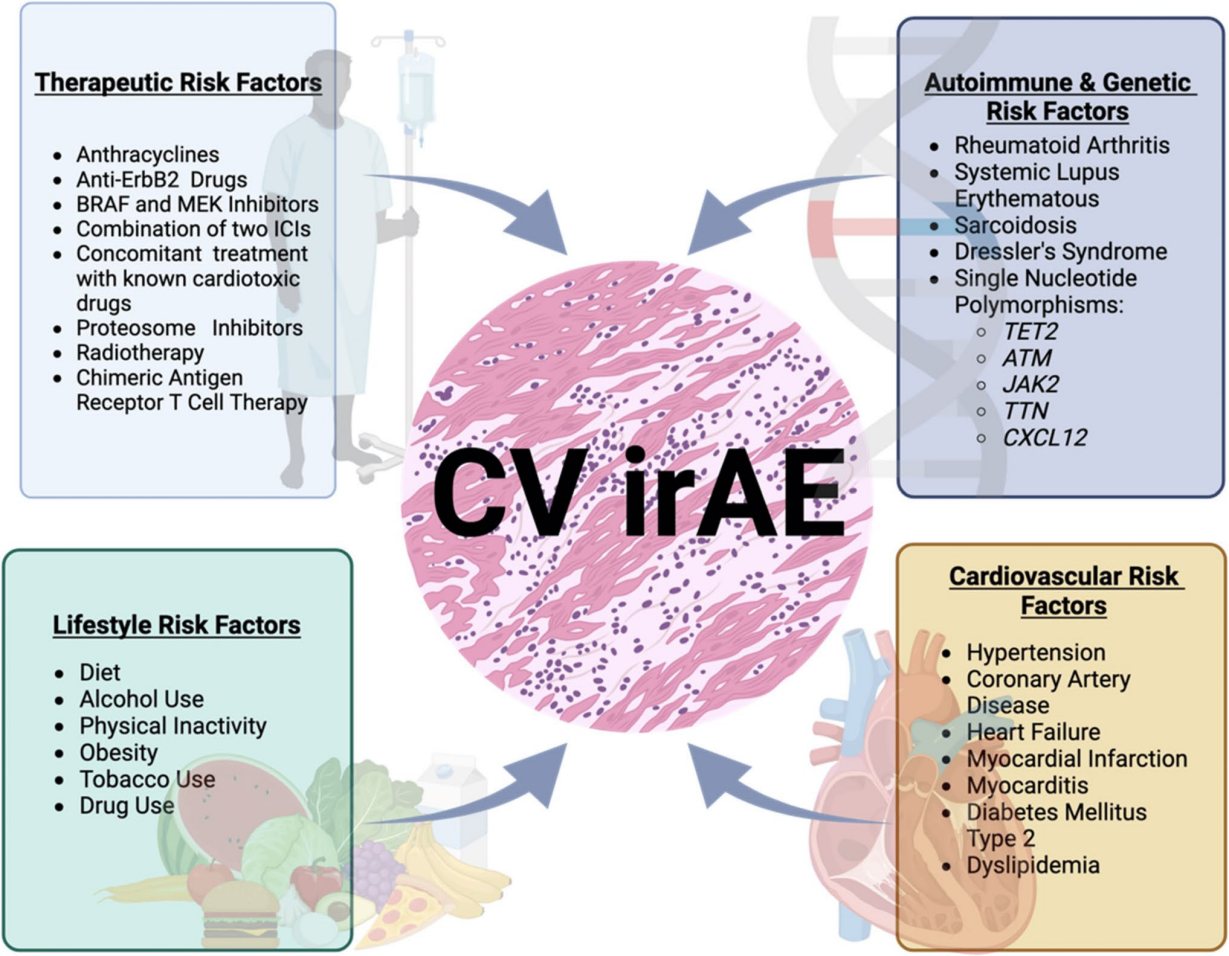
Patient-related Factors and ICI Cardiotoxicity. While the toxic effects of immune checkpoint inhibitors can be seen in a broad range of patients and cancers, there are patient-related factors that portend a higher risk. Combination therapy with immune checkpoint inhibitors (ICIs) and other agents—including BRAF/MEK inhibitors, tyrosine kinase inhibitors, radiation, and chimeric antigen receptor T-cell (CAR-T) therapy—has been associated with increased risk or potentially additive risk of cardiovascular immune-related adverse events, including myocarditis and other cardiotoxicities. The risk appears particularly elevated with ICI combination regimens and when ICIs are used alongside targeted therapies such as BRAF/MEK inhibitors or tyrosine kinase inhibitors, as these agents have independent cardiotoxic profiles and may have synergistic effects on cardiac risk. CAR-T cell therapy is also associated with cardiac toxicity, primarily in the context of cytokine release syndrome, and may further increase risk when combined with ICIs. Radiation, particularly thoracic radiotherapy, may have additive or synergistic effects with ICIs in increasing cardiac toxicity, but definitive data on the magnitude of this risk are still emerging. Preclinical and clinical data suggest overlapping mechanisms of injury, and combination regimens should be approached with caution. From: Green, C.E.; Chacon, J.; Godinich, B.M.; Hock, R.; Kiesewetter, M.; Raynor, M.; Marwaha, K.; Maharaj, S.; Holland, N. The Heart of the Matter: Immune Checkpoint Inhibitors and Immune-Related Adverse Events on the Cardiovascular System. *Cancers* 2023, *15*, 5707. 10.3390/cancers15245707

The pathogenesis of ICI-associated cardiotoxicity has been summarized previously. In brief, myocarditis is primarily driven by the infiltration of cytotoxic CD8 + T cells into the myocardium. These T cells recognize cardiac antigens, leading to direct myocardial injury. Studies have shown an expansion of clonally activated CD8 + T cells with a cytotoxic phenotype in patients with ICI myocarditis [49]. ICIs can promote macrophage polarization towards a pro-inflammatory M1 phenotype via the cGAS-STING pathway and provoke the release of proinflammatory cytokines such as IL-17A, TNF-α, and IFN-γ [65] (Fig. 3).

By contrast, dermatologic and gastrointestinal toxicities are often mediated by different immune pathways, including the activation of Th1 and Th17 cells, while endocrine toxicity is often autoimmune in nature with activation of autoreactive T cells and the production of autoantibodies [66]. Antigens for toxicity are also tissue specific.

### Cardiovascular antigens involved in ICI-associated toxicity

Antigen targets for ICI cardiotoxicity encompass various cardiac proteins and cells. Heavy chain proteins, which are components of antibodies, can become targets when ICs are inhibited, leading to an autoimmune response. A notable example is the α-myosin heavy chain, a key structural protein in cardiomyocytes. Remnant thymic cells, involved in the development of immune tolerance, may also be implicated as they can present self-antigens that trigger an immune attack on the heart [67]. In addition to heavy chain proteins and remnant thymic cells, several other specific antigens in the heart can be targeted during ICI cardiotoxicity. These include the ubiquitous proteins troponin and actin. Understanding these specific antigens is crucial for identifying the mechanisms of ICI-induced cardiotoxicity and developing targeted therapies to mitigate these adverse effects [68]. A detailed summary of cardiac immunology, including central and peripheral immune tolerance is summarized in Koc and Vicenzetto [25, 69].

### Myotoxicity spectrum

The *spectrum of myotoxicity* observed with ICIs includes myositis, myocarditis, myasthenia gravis, neuromyopathy, and, less commonly, rhabdomyolysis and fasciitis. Myositis is the most frequently reported form, often presenting with proximal muscle weakness, myalgia, and elevated creatine kinase, and is notable for frequent overlap with myocarditis and myasthenia gravis, which significantly increases morbidity and mortality [70]. The pathophysiology is characterized by T cell–mediated cytotoxicity against muscle and cardiac antigens, with single-cell RNA sequencing and tissue studies in humans revealing infiltrates of cytotoxic CD8 + T cells, myeloid cells expressing FcγRIIIa, and complement activation, sometimes accompanied by high-titer autoantibodies. Thymic alterations and pre-existing autoimmunity further increase susceptibility, as shown in both clinical registries and pharmacovigilance data (reviewed in Hamada e. al.) [71, 72].

ICI-associated myotoxicity is rare, with an incidence of myositis around 0.38% in phase III trials, but the risk is higher with combination therapy and in older patients. Myotoxicity typically occurs within the first three months of ICI initiation, often after the first or second dose, and can progress rapidly to life-threatening complications, especially when myocarditis or bulbar/respiratory involvement is present. While muscle enzyme levels (CK) often normalize within weeks of immunosuppression, cardiac biomarkers (troponin) may remain elevated for months, and the acute phase carries a high risk of fatal arrhythmias and respiratory failure [73].

Understanding the full spectrum of ICI-associated myotoxicity—its clinical overlap, rapid onset, and immune-mediated mechanisms—has clarified that ICI toxicity reflects a systemic breakdown of self-tolerance, often with concurrent cardiac and neuromuscular involvement [74]. It also underscores the need for early detection, multidisciplinary management, and the development of next-generation immunotherapies with improved selectivity to minimize off-target immune activation.

### Cancer type and cardiotoxicity

The type of cancer being treated can also influence the incidence of ICI-myocarditis. For a detailed examination of the association between tumor type, ICI therapy, and other immune-related adverse events, a recent study using a worldwide pharmacovigilance database is recommended. This analysis of over 140,000 cases of irAEs from ICI therapy found that thymic epithelial tumors (TETs) were associated with an increased odds ratio of 23.56 (95% CI 15–38) for ICI-myocarditis [75].

### Patient-specific genetic and epi-genetic factors and cardiotoxicity

Cardiovascular toxicity from immunotherapy can vary from person to person because of several factors. Genetic differences play a crucial role because inherent variations impact treatment response, with some individuals having a predisposition to cardiovascular toxicity. For example, HLA-DRB1*11:01 has been linked to higher toxicity in patients treated with ICIs, polymorphisms in CTLA-4 have been associated with increased risk of autoimmune toxicity, aberrant methylation patterns in immune-related genes have been implicated in the development of ICI toxicity, miRNAs have been identified as potential biomarkers for predicting ICI toxicity, and genetic variations that affect cytokine production [76, 77] and signaling can also contribute to toxicity-most often via interleukin (IL)-6 production [78].

Several studies have identified genetic variants associated with an increased risk of irAEs, including cardiotoxicity, in patients receiving ICIs. For example, a genome-wide association study identified significant associations with variants near IL7, IL22RA1, and on chromosome 4p15, which were linked to all-grade irAEs [79]. Specifically, the rs16906115 variant near IL7 was associated with increased lymphocyte stability after ICI initiation, which is predictive of downstream irAEs and improved survival.

There may also be an important association between specific immune-related genes and ICI-related myocarditis. Key genes such as IL7R, PRF1, GNLY, CD3G, NKG7, GZMH, GZMB, KLRB1, KLRK1, and CD247 have been identified as pivotal in the development of ICI-associated myocarditis [80]. These genes are involved in pathways related to cell lysis, CD8 + T-cell receptor signaling, and natural killer cell-mediated cytotoxicity.

Variants in genes such as TET2, ATM, JAK2, TTN, and CXCL12 (see Fig. 3) have been implicated in modulating immune responses, inflammation, and cardiac structural integrity, each of which are relevant to the pathogenesis of ICI-related cardiac adverse events. For example, pathogenic variants in TTN, a gene encoding a key sarcomeric protein, are established risk factors for dilated cardiomyopathy and may predispose to cardiac injury under additional stressors such as ICI therapy. Similarly, mutations in TET2, ATM, and JAK2 are associated with clonal hematopoiesis and heightened systemic inflammation, which could amplify immune-mediated cardiac toxicity in the context of ICI exposure. CXCL12, a chemokine involved in immune cell trafficking, may also influence the degree of immune infiltration and inflammation in cardiac tissue during ICI therapy Reviewed in Kim) [81]. However, the available literature does not establish a direct *causal* relationship for any single gene, nor does it provide clarity of whether the gene- drug interactions or associations are *synergistic* or *additive* effects among these genetic factors in the context of ICI cardiotoxicity. Larger, mechanistic investigations are needed to clarify these interactions [82].

### Other patient- and treatment- related factors

Patient-related factors associated with increased risk for ICI-associated myocarditis and vasculitis include those receiving dual (or bi-specific) ICI therapy, older adults, men, and people with a history of arrhythmias, lung cancer, or central nervous system cancers [83]. Dual ICI therapy significantly increases the risk of myocarditis and vasculitis, with studies suggesting a 2.0-fold increased risk of occurrence and a 2.9-fold increase in mortality associated with these conditions [84]. In addition, patients with pre-existing cardiovascular conditions, hypertension, diabetes mellitus or those undergoing concurrent anti-VEGF therapy are at heightened risk [85]. These findings underscore the importance of careful cardiovascular risk assessment and monitoring in patients being considered for and those undergoing ICI therapy [86].

Combined cancer therapies to include BRAF/MEK inhibitors, TKI, radiation, and CAR-T cell therapy, drugs and modalities that carry their own risk for cardiotoxicity may be risk factors for ICI-related cardiotoxicity. They may also have synergistic effects or additive effects. Most available data are from case series, retrospective reviews, or meta-analyses, and there is a lack of large prospective studies directly quantifying the additive risk of these combinations; further research is needed to clarify these relationships [87] [88] (see Fig. 3).

## Frequency of ICI induced cardiac toxicity

The incidence of cardiovascular adverse events (CVAEs) associated with ICI’s ranges from less than 1% to approximately 18%, depending on the specific ICI and patient population [89]. Myocarditis, a severe form of cardiotoxicity, typically occurs early after the initiation of ICI therapy, with a median onset time of 2 months and most cases occurring within the first 3 months [90]; [91]. However, cardiovascular toxicity can manifest at any time during ICI therapy and may occasionally occur even after the cessation of therapy due to the persistent effects of the drug.

The incidence of myocarditis is between 0.24% and 1%, and it can be fatal. While the left ventricle is primarily involved in most cases, myocarditis localized to the right ventricle has also been reported [92–94]. A summary of cardiotoxicities and non-cardiac toxicities that could mask, augment, or less commonly attenuate the cardiovascular effects is shown in Table 2.

## Cardiotoxicity profiles of chemotherapy and immune check point inhibitor drugs

Anthracyclines and ICIs are used in combination for some cancer treatments, particularly in advanced solid tumors such as breast cancer and non-small cell lung cancer, where this approach is now a standard of care in certain settings. The rationale for combining these therapies is based on the ability of anthracyclines (e.g., doxorubicin) to induce immunogenic cell death, thereby enhancing tumor antigen presentation and priming the immune system for a more robust response to ICIs. This synergy has been demonstrated in both preclinical and clinical studies, leading to improved outcomes in several tumor types [95, 96].

It is also common for these therapies to be given sequentially, such as administering chemotherapy first and then switching to ICIs upon disease progression or as maintenance therapy. However, there are no head-to-head trials directly comparing sequential versus concurrent administration, and the optimal sequencing remains an area of active investigation. Some studies suggest that maintenance ICI after chemotherapy may provide additional benefit in certain cancers, but more data is needed to define best practices, especially for toxicity and long-term outcomes. The potential for facilitated ICI toxicity must also be clarified.

Specifically, the findings of Kortse and colleagues that suggest PD1/PDL1 signaling is affected after anthracycline treatment, potentially contributing to an increased susceptibility to immune-related adverse events of subsequent anti-PD1/PDL1 cancer therapy requires further investigation [97].

### Distinguishing features

Chemotherapy-associated cardiotoxicity and ICI cardiotoxicity differ significantly in their clinical presentation, time to onset, imaging findings, and natural history upon cessation of therapy. For example, doxorubicin-associated cardiotoxicity can present acutely (within 1 week of initiation), early (within 1 year), or late (years to decades later). ICI cardiotoxicity typically presents early, with a median onset of 34 days after initiation of therapy but can range from 2 to 54 weeks [98].

Chemotherapy-associated cardiotoxicity often manifests as heart failure, with symptoms including dyspnea, fatigue, and peripheral edema. Acute presentations can include arrhythmias and cardiogenic shock. By contrast, ICI cardiotoxicity can present with myocarditis, pericarditis, arrhythmias, and heart failure. Symptoms include fatigue, myalgia, chest pain, palpitations, and in severe cases, cardiogenic shock or sudden death [45].

Chemotherapy-associated cardiotoxicity may partially or completely recover if detected early and managed appropriately, but some patients may develop chronic heart failure [99]. ICI cardiotoxicity often requires discontinuation of the offending agent. Immediate recognition and optimal management can result in a steady improvement, but some patients have persistent or progressive cardiac dysfunction [100].

## Screening strategies for ICI-related cardiotoxicity

**Fig. 4:**
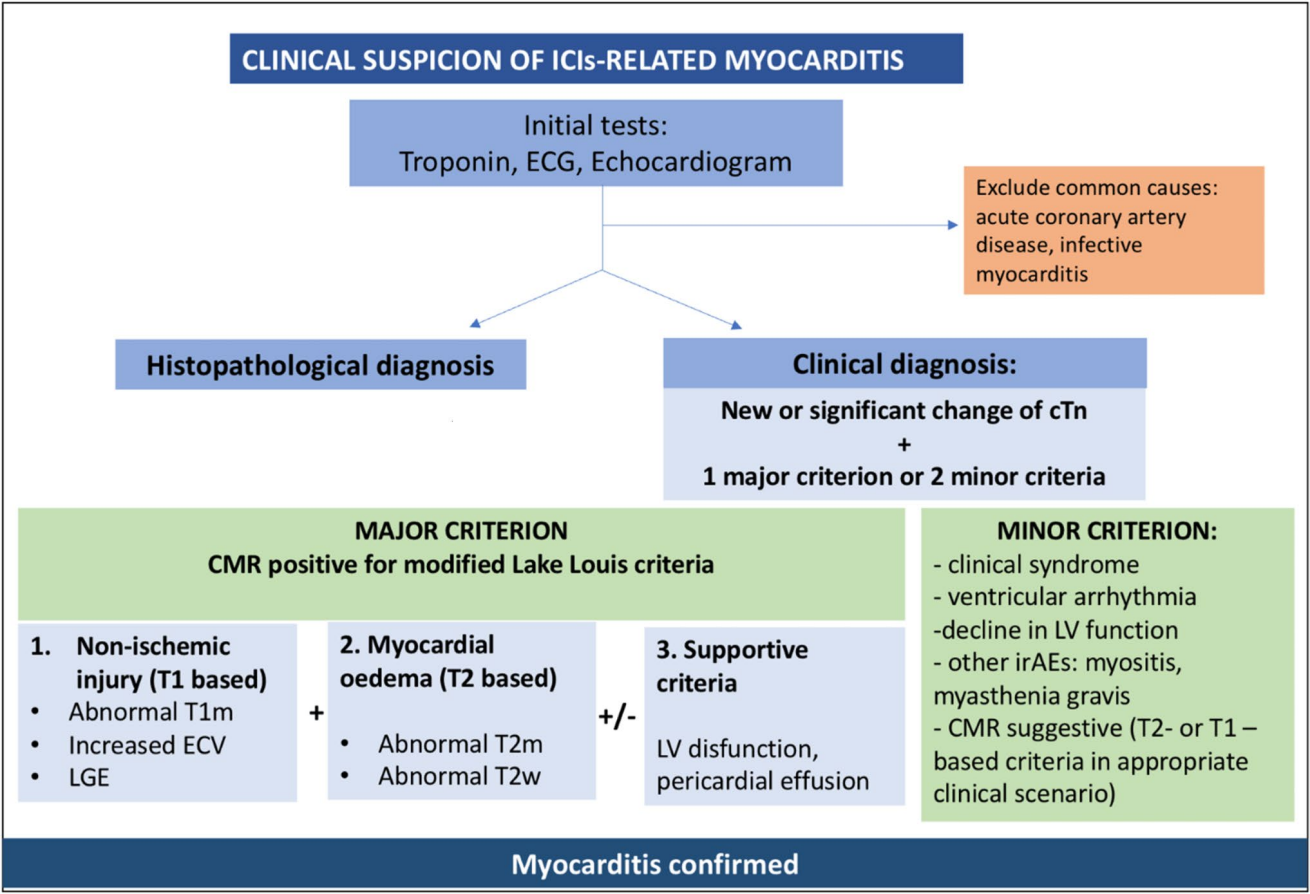
Screening Strategies to Detect ICI Cardiotoxicity. An early diagnosis of ICI-related cardiotoxicity is required for optimal management and outcomes. Baseline studies including an electrocardiogram, echocardiogram, brain natriuretic peptide (BNP) and high sensitivity troponin (hsTN) provide an important reference point or comparator for changes that might occur during treatment. Cardiac MRI is an important diagnostic modality. Major and minor criteria are important when the clinical suspicion is high. From: Frascaro, F.; Bianchi, N.; Sanguettoli, F.; Marchini, F.; Meossi, S.; Zanarelli, L.; Tonet, E.; Serenelli, M.; Guardigli, G.; Campo, G.; et al. Immune Checkpoint Inhibitors-Associated Myocarditis: Diagnosis, Treatment and Current Status on Rechallenge. *J. Clin. Med.* 2023, *12*, 7737. 10.3390/jcm12247737

Screening patients for potential cardiovascular toxicity before starting ICIs and monitoring them during treatment is crucial due to the risk of CVAEs, including myocarditis. Before starting treatment, practitioners must conduct a thorough baseline cardiac risk assessment, which involves evaluating the patient’s cardiovascular history, including any previous heart conditions, hypertension, diabetes, and family history of heart disease. Baseline tests such as an electrocardiogram (ECG) are performed to detect any pre-existing cardiac abnormalities, while an echocardiogram assesses cardiac function and structure. Measuring cardiac biomarkers like troponin and natriuretic peptides (BNP or NT-proBNP and high-sensitivity troponin) helps identify any underlying cardiac stress or damage. For patients with high cardiovascular risk or existing heart conditions, involving a cardiologist with expertise in cardio-oncology is recommended (Fig. 4).

During treatment, regular cardiac biomarker testing is essential, with measurements taken before each of the first three cycles of ICI therapy and periodically thereafter. ECG monitoring should be conducted at regular intervals (every 12 weeks) to detect any new cardiac abnormalities, and echocardiograms should be repeated periodically (every 12 weeks), especially if symptoms are suggestive of cardiac complications. A cardiac MRI (Magnetic Resonance Imaging) is considered a gold standard for securing a diagnosis of ICI-related myocarditis [101–104].

Patients must be educated and reminded to report symptoms such as chest pain, shortness of breath, palpitations, or fatigue immediately.

### Does the troponin assay matter in cardiotoxicity surveillance?

High-sensitivity cardiac troponin T (hs-cTnT) is the preferred assay for screening for ICI-associated myocarditis due to its superior sensitivity for early detection of myocardial injury, strong association with major adverse events, and ability to identify at-risk patients, including those with overlapping myositis or myasthenia gravis syndromes. Serial monitoring of hs-cTnT before and during ICI therapy, particularly in high-risk populations such as patients with thymic epithelial tumors, those receiving combination ICI therapy, and older adults, enables early identification and intervention, which can reduce morbidity and mortality. The rationale for choosing hs-cTnT over troponin I is its higher sensitivity in detecting both cardiac [105] and skeletal muscle injury, which is relevant given the T cell–mediated cytotoxicity affecting both myocardium and skeletal muscle in ICI toxicity; however, clinicians should be aware that hs-cTnT may be elevated in isolated myositis, potentially leading to false positives in the absence of myocarditis [106].

A substantial proportion of clinical laboratories in the US- estimated at over two-thirds use only troponin I assays. In the European Union (EU), adoption of high-sensitivity troponin T assays is more widespread, but there remains significant heterogeneity, with many laboratories still using only troponin I assays due to local vendor contracts and regulatory approvals [107]. Reliance on troponin I alone may result in missed or delayed diagnosis of ICI myocarditis, particularly in cases with mild or subclinical myocardial injury, and may reduce the ability to risk-stratify patients for adverse outcomes [108]. This gap underscores the need for harmonization of assay availability and the development of standardized protocols for ICI myocarditis surveillance, as recommended by the ACC [105].

## Management of ICI associated cardiotoxicity

The treatment of ICI-related cardiotoxicity in 2025 involves a multi-faceted approach, starting with high-dose corticosteroids as the first-line therapy. For refractory cases, additional immunosuppressive agents should be considered. Standard heart failure management protocols should be followed, and discontinuation of ICI therapy is essential. Supportive care measures are also critical, and emerging therapies targeting the NLRP3 inflammasome show promise in reducing cardiotoxicity [109, 110].

The ESC and the ACC provide comprehensive guidelines for managing ICI associated cardiac toxicity [111]. A comprehensive summary of ICI toxicity by system and management was drafted by the ASCO [45] (Table 3). By following these guidelines, healthcare practitioners can effectively manage ICI-induced cardiotoxicity, ensuring timely intervention and improved patient outcomes [112].

**Table 3:** Management of immune checkpoint-induced cardiotoxicity

Treatment	Description
High-dose corticosteroids	Initial treatment with high-dose corticosteroids (e.g., 1000 mg of methylprednisolone IV daily for 3–5 days) is recommended for suspected or confirmed ICI-related myocarditis
Second-line immunosuppressive therapy	For refractory cases, additional immunosuppressive agents such as mycophenolate mofetil, infliximab, or abatacept may be used
Standard heart failure management	In cases of heart failure, standard heart failure treatments including beta-blockers, ACE inhibitors, and diuretics should be initiated
Discontinuation of ICI therapy	Immediate cessation of ICI therapy is crucial upon diagnosis of myocarditis
Supportive care	Includes measures such as fluid resuscitation, vasopressors for hemodynamic support, and mechanical circulatory support if needed
NLRP3 inflammasome inhibitors	Emerging evidence suggests that targeting the NLRP3 inflammasome with agents like MCC950 may reduce cardiotoxicity and improve outcomes

American society of clinical oncology ICI cardiotoxicity management	European society of cardiology ICI cardiotoxicity management
• Early Identification and Monitoring: Clinicians should maintain a high level of suspicion for cardiotoxicity in patients presenting with new cardiovascular symptoms • Diagnostic Evaluation: At symptom presentation, an echocardiogram should be performed to assess ventricular function. Cardiac MRI can be used to detect myocarditis, though it is less sensitive than endomyocardial biopsy, which should be considered in unstable patients or those not responding to initial therapy	• Early Detection and Monitoring: Baseline cardiovascular assessment, including history, physical examination, ECG, and troponin levels, is recommended before initiating ICI therapy. Regular monitoring of cardiac biomarkers and ECGs during treatment is advised • Diagnostic Evaluation: In patients presenting with symptoms suggestive of myocarditis, the ESC recommends immediate cessation of ICI therapy and prompt initiation of diagnostic workup, including ECG, troponin levels, echocardiography, and cardiac MRI. Endomyocardial biopsy should be considered in cases where the diagnosis is uncertain or in severe cases
Management of Cardiotoxicity: • Grade 1 Toxicities: Continue ICPi therapy with close monitoring • Grade 2 Toxicities: Hold ICPi therapy and consider corticosteroids (0.5–1 mg/kg/day of prednisone or equivalent) until symptoms revert to ≤ grade 1 • Grade 3 Toxicities: Hold ICPi therapy and initiate high-dose corticosteroids (1–2 mg/kg/day of prednisone or equivalent) [1]	Management of Myocarditis: • Mild Cases (Grade 1–2): Hold ICI therapy and initiate corticosteroids at a dose of 1–2 mg/kg/day of prednisone or equivalent. Taper corticosteroids over 4–6 weeks once symptoms improve and biomarkers normalize • Severe Cases (Grade 3–4): High-dose corticosteroids (1–2 mg/kg/day of methylprednisolone) should be administered. If there is no improvement within 24–48 h, additional immunosuppressive therapies may be considered [2]
Cardiology Involvement: Early involvement of cardiology and continuous telemetry monitoring are recommended due to the risk of arrhythmias and progression to life-threatening conditions	Rechallenge Considerations: Rechallenge with ICIs after resolution of myocarditis should be approached with caution. The decision should be individualized, involving a thorough risk–benefit analysis and multidisciplinary team discussion. Close monitoring is essential if rechallenge is pursued [3]
Empirical Treatment: In cases of high clinical suspicion of myocarditis, empirical treatment should be initiated before confirmatory testing	Supportive Care: Standard heart failure therapies should be employed in patients with reduced ejection fraction. Arrhythmias should be managed according to standard guidelines, and patients with severe myocarditis may require mechanical circulatory support

*ICPi* Immune checkpoint inhibitor

When cardiotoxicity is suspected, immediate steps include discontinuing ICI therapy and conducting baseline tests (hs-TnI or TnT and BNP) and performing an ECG and a transthoracic echocardiogram [113]. Confirmatory tests like cardiac MRI and endomyocardial biopsy should be considered if the diagnosis is uncertain.

Treatment includes administering corticosteroids, with oral prednisone for mild to moderate cases and intravenous methylprednisolone for severe cases, continuing until symptoms resolve and cardiac biomarkers normalize, followed by a taper over 4–6 weeks [114]. Additional immunosuppressive therapy should be considered for refractory cases, though infliximab is contraindicated in patients with heart failure [93, 115].

The management of heart failure includes discontinuation of ICI therapy and initiation of high-dose corticosteroids. Standard heart failure therapies, including beta-blockers, ACE inhibitors, or ARBs, sacubitril/valsartan, mineralocorticoid receptor antagonists and diuretics, should be employed as needed. A cardiology consultation is essential for ongoing management [116, 117].

### Advanced medical therapies

Extracorporeal membrane oxygenation (ECMO) can be used in cases of fulminant myocarditis or severe ventricular dysfunction refractory to medical therapy. The International Society for Heart and Lung Transplantation and the Heart Failure Society of America recommend ECMO for patients with severe cardiogenic shock to provide biventricular unloading, maintain systemic and coronary perfusion, and prevent multiorgan dysfunction. Veno-arterial ECMO (VA-ECMO) is particularly useful in stabilizing hemodynamics and providing a bridge to recovery, transplantation, or durable mechanical support [118].

Left ventricular assist devices (LVADs) can be considered when prolonged mechanical support is necessary. In cases where ECMO alone is insufficient, combining ECMO with a percutaneous LVAD, such as the Impella device, can enhance left ventricular unloading and promote myocardial recovery. This combination, sometimes referred to as “ECMELLA,” has shown efficacy in managing severe myocarditis and cardiogenic shock by providing the necessary time for diagnostics and specific therapy initiation [119].

The American Society of Clinical Oncology (ASCO) guidelines also emphasize the importance of mechanical circulatory support in managing severe cardiovascular toxicities from ICIs, including myocarditis and cardiogenic shock [45].

The management of pericarditis involves holding ICI therapy and starting corticosteroids (prednisone 1–2 mg/kg/day or equivalent). Nonsteroidal anti-inflammatory drugs (NSAIDs) and colchicine may be used for symptom control.

For conduction abnormalities and arrhythmias, ICI therapy should be discontinued, and corticosteroids should be initiated. Continuous telemetry monitoring and early cardiology involvement are crucial. Management of specific arrhythmias follows standard protocols, including antiarrhythmic drugs and potential use of devices like pacemakers or defibrillators for severe cases. There is mounting evidence for sacubitril/valsartan having anti-inflammatory and anti-arrhythmic effects [120].

## Short-term outcomes

The short-term mortality rate for ICI-associated myocarditis is alarmingly high, ranging from 27 to 60% [121]. Most patients with severe myocarditis require hospitalization, often in the intensive care unit due to the risk of cardiogenic shock and other severe complications [122].

## Long-term outcomes

Approximately 50% of patients who survive the initial myocarditis episode may develop chronic heart failure. Ventricular tachycardia (VT) and VT storm are significant long-term risks, contributing to morbidity and mortality. Cardiogenic shock is a severe complication that can occur both in the short and long term, often requiring mechanical circulatory support. Long-term mortality remains high, with up to 48% of patients either dying or requiring heart transplantation within seven years [123].

## Other cardiovascular complications of ICI therapy

Immune checkpoint inhibitor (ICI)-related cardiac toxicity encompasses a spectrum of clinical phenotypes as summarized below.

Takotsubo cardiomyopathy (TTC), also known as stress-induced cardiomyopathy, has been reported in patients undergoing ICI therapy. Immune-related adverse events (irAEs), including cardiovascular toxicities. A systematic review by Trontzas et al. identified 17 cases of TTC in cancer patients treated with ICIs, with a median time to presentation of 77 days after starting therapy. The most frequently implicated agents were pembrolizumab and the combination of nivolumab and ipilimumab [124]. The review highlighted that most patients recovered from TTC, although some experienced relapses or concurrent cardiac toxicity. Management of TTC typically involves supportive care, including beta-blockers and ACE inhibitors, and in some cases, corticosteroids may be used to manage the underlying inflammatory response [45].

The myocardium in the left and right atria is relatively thin compared to the ventricles. The left atrium has a slightly thicker myocardium than the right atrium due to the higher pressure generated when receiving oxygenated blood from the lungs. Atrial myopathy can occur with several cardiovascular toxicities, including myocarditis and non-inflammatory dysfunction and contribute to atrial arrhythmias (atrial fibrillation and atrial flutter), endocardial or appendage thrombosis, and cardioembolic events [125, 126].

Mural thrombosis, the formation of a thrombus, is most often on the endocardial surface of the left ventricle or right ventricle can occur as a complication of myocarditis or from impaired cardiac contractility in the absence of myocarditis. The immuno-inflammation and damage to the myocardial tissue create a pro-thrombotic environment, increasing the risk of thrombus formation [127]. The collective mechanisms are as follows- left ventricular dysfunction and stasis: reduced contractility of the left ventricle leads to blood stasis, which is an important factor in thrombus formation; endocardial injury: damage to the endocardial lining of the heart creates a highly pro-thrombotic surface; and hypercoagulability: an increased tendency for blood to clot, which in the setting of ICI toxicity can be due to systemic inflammation. These elements are part of Virchow's triad, which describes the conditions that predispose to thrombosis [128, 129].This can lead to further complications such as embolic events, including visceral or peripheral artery occlusion, stroke or myocardial infarction. Systemic anticoagulant therapy is recommended for a minimum of 12 weeks in the absence of a thromboembolic event and for up to a year if a thromboembolic event has occurred. Current guidelines support either vitamin K antagonist or direct oral anticoagulants (DOACS) [130].

Conduction and arrhythmogenic toxicities associated with ICIs include complete heart block and a variety of dysrhythmias, such as supraventricular tachycardias and life-threatening ventricular tachycardias. These toxicities can but less commonly occur in the absence of myocarditis.

The American Society of Clinical Oncology (ASCO) guidelines highlight that cardiovascular irAEs from ICIs can present as conduction abnormalities and arrhythmias independently of myocarditis [45]. The conduction system and arrhythmogenic toxicities can occur due to direct lymphocytic infiltration [64]. A comprehensive analysis of the FDA Adverse Event Reporting System highlighted that cardiac arrhythmias are a significant concern with ICIs, with anti-PD-1 and anti-PD-L1 therapies being associated with higher reports of arrhythmias compared to anti-CTLA-4 therapies. This includes both supraventricular and ventricular arrhythmias, which can occur without concurrent myocarditis [131].

## Vascular complications

Vascular complications associated with ICIs include both short-term and long-term risks, with evidence from original research in human populations. In the short term, ICIs are associated with a significantly increased risk of arterial thrombotic events such as myocardial infarction and ischemic stroke, as well as venous thrombosis (see section below). For example, a cohort study demonstrated a threefold increased risk of myocardial infarction and a 3.2-fold increased risk of ischemic stroke in patients receiving ICIs compared to the general population, with these events occurring during and shortly after treatment initiation. Another large, matched cohort and case-crossover study found a 3- to 4.8-fold increased risk of atherosclerotic cardiovascular events within two years of ICI initiation, with the incidence rising from 1.37 to 6.55 per 100 person-years [132].

There is evidence that ICIs accelerate atherogenesis. Imaging sub-study data show that the rate of aortic plaque progression is more than threefold higher after starting ICIs, suggesting that these agents may promote the development and progression of atherosclerotic disease beyond the immediate treatment window [133].The increased risk of both acute vascular events and accelerated plaque progression underscores the need for ongoing cardiovascular risk assessment in cancer survivors treated with ICIs.

Retrospective and meta-analytic data indicate that statin use during or after ICI initiation is associated with lower rates of major adverse cardiovascular events and improved overall/progression-free survival, with some evidence suggesting greater benefit when statins are started after ICI initiation and with lipophilic statins [134]. There are no formal guidelines on the timing of statin initiation in ICI-treated patients, but available data support starting statins in those with traditional cardiovascular risk factors or evidence of ICI-induced cardiovascular toxicity, and possibly considering statins for broader primary prevention in this population [135]; further prospective studies are needed to clarify optimal timing and patient selection.

### Arterial and venous thrombosis

Venous and arterial thrombosis associated with ICIs can be attributed to several mechanisms, including T-cell activation and anti-tumor immunity. This heightened immune response can lead to systemic inflammation, which is a known risk factor for thrombosis. Elevated levels of inflammatory cytokines, such as IFN-γ, TNF-α, IL-1β, IL-6, and IL-17, contribute to endothelial dysfunction, plaque destabilization, and thrombosis [52]. The disruption of immune homeostasis by ICIs can lead to autoimmune-mediated inflammation of the vascular endothelium. This inflammation can result in endothelial injury, promoting both venous and arterial thrombosis [136].

Metabolic changes in cancer cells and immune cells are believed to be contributors to a pro-thrombotic state. Metabolic remodeling involving acetate, pyruvate, and amino acids have been observed in non-small cell cancer cells exposed to ICIs, which may promote coagulation [137].

Immune checkpoint inhibitors can induce the expression of tissue factor (TF) on monocytes and macrophages, which promote a thrombophilia state. This particular property is clinically relevant in cancer patients, where the interaction between cancer cells, immune cells, and ICIs can potentiate coagulation [137].

Although direct effects of ICIs on platelet activation are not well-established, the indirect effects through immune-mediated pathways are likely a major contributor. ICIs can lead to the release of pro-thrombogenic factors from immune cells, which in turn can activate platelets and promote thrombus formation [138].

The treatment of thrombosis that occurs during ICI treatment should include anticoagulants chosen according to clinical judgement, renal performance, preferred drug half-life, and other considerations. In the absence of signs or symptoms of toxicity affecting one or more organs, ICI cessation may not be required because many of the cancers treated with ICIs can themselves be associated with thrombosis (Table 4).

**Table 4:** Immune checkpoint inhibitor-induced vasculitis

Vessel size	Types of vasculitis	Clinical features	Management
Small Vessel	IgA vasculitis Cryoglobulinemic vasculitis ANCA-associated vasculitis Unclassified small vessel vasculitis	Cutaneous involvement Renal involvement Neurological manifestations	Discontinuation of ICI Systemic corticosteroids Additional immunosuppressive agents
Medium Vessel	Polyarteritis nodosa Renal vasculitis	Skin findings Renal involvement Systemic symptoms (fever, weight loss)	Discontinuation of ICI Systemic corticosteroids Additional immunosuppressive agents (e.g., cyclophosphamide)
Large Vessel	Giant cell arteritis Takayasu arteritis	Constitutional symptoms (fever, weight loss) Limb claudication Visual disturbances Aneurysm Formation Aortic or Branch Vessel Dissection	Discontinuation of ICI High-dose corticosteroids Additional immunosuppressive agents (e.g., tocilizumab)

## Immune check point inhibitor-induced vasculitis

Programmed cell death protein 1 (PD-1) and PD-L1 are specific vascular antigens that are targets for immune checkpoint inhibitor-induced vasculitis. For instance, in giant cell arteritis (GCA), a deficiency in PD-L1 expression on dendritic cells and macrophages within the vessel wall allows PD-1 positive T cells to infiltrate and facilitate. Similarly, in ANCA-associated renal vasculitis, a loss of PD-1 expression correlates with active disease [139].

Vasculitis, an inflammation of blood vessels, can affect various vascular beds including large vessels (e.g., aorta and its branches), medium vessels (e.g., coronary and renal arteries), and small vessels (e.g., capillaries, venules, arterioles) [140]. Each has been described in ICI-related vascular toxicity. Complications can include aortic aneurysms, aortic dissection, visual loss, mononeuritis, amaurosis fugax and clinical features of thrombosis, which can lead to organ ischemia and infarction [141–143] (Table 5).

**Table 5:** Immune checkpoint inhibitor-induced vasculitis

Vessel size	Types of vasculitis	Clinical features	Management
Small vessel	IgA vasculitis Cryoglobulinemic vasculitis ANCA-associated vasculitis	Cutaneous involvement Renal involvement Neurological manifestations	Discontinuation of ICI Systemic corticosteroids Additional immunosuppressive agents
Medium vessel	Unclassified small vessel vasculitis Polyarteritis nodosa Renal vasculitis	Skin findings Renal involvement Systemic symptoms (fever, weight loss)	Discontinuation of ICI Systemic corticosteroids Additional immunosuppressive agents (e.g., cyclophosphamide)
Large vessel	Giant cell arteritis Takayasu arteritis	Constitutional symptoms (fever, weight loss) Limb claudication Visual disturbances Aneurysm Formation Aortic or Branch Vessel Dissection	Discontinuation of ICI High-dose corticosteroids Additional immunosuppressive agents (e.g., tocilizumab)

### Vasculitis and thrombosis

Endothelial cell inflammation and associated glycocalyx injury create a highly prothrombotic environment that can affect both microvascular and macrovascular beds. The microvascular circulatory system consists of arterioles, capillaries, and venules. Arterioles have a primary role in regulating distribution of blood flow, while the capillaries represent the primary site of fluid and solute exchange, and the venules are the primary site of interaction with immune cells. The capillary beds do not hold a significant portion of the blood volume—estimated at less than 10% of the total volume—but have an enormous surface area for exchange (reviewed in Bray) [144]. Thrombosis has been defined mechanistically as the end-result of impaired biophysical properties of erythrocytes and leukocytes to include decreased deformability, heightened cell–cell interactions, soluble factors and perturbed or dysfunctional endothelial cells characterized by a loss of protective cell-surface anticoagulant, anti-platelet, fibrinolytic and anti-leukocyte proteins. Inflammation and both leukocyte and platelet-rich thrombi in a shear-stress dependent environment are the pathophysiological hallmarks of microvascular thrombosis [145].

### Proposed pathogenesis of vasculitis

The target antigens are often components of the vascular endothelium [146]. The immune response is typically directed against endothelial cells, leading to inflammation and damage of the blood vessel walls. This process involves the activation of T-cells and the release of pro-inflammatory cytokines, which contribute to endothelial injury and subsequent vasculitis [147]. A list of the target antigens is summarized below.

- Vascular Endothelial Growth Factor Receptor (VEGFR): This receptor is crucial for angiogenesis and endothelial cell function.
- Intercellular Adhesion Molecule-1 (ICAM-1): ICAM-1 plays a role in the adhesion of leukocytes to endothelial cells, facilitating their migration into tissues.
- Endothelial Cell-Specific Molecule-1 (ESM-1): Also known as endocan, this molecule is involved in the regulation of vascular permeability and leukocyte migration.
- Platelet Endothelial Cell Adhesion Molecule-1 (PECAM-1): PECAM-1 is important for leukocyte transmigration and maintaining endothelial cell junctions.
- CD93: This molecule is involved in cell adhesion and angiogenesis and has been identified as a target in endothelial cells during inflammatory responses.

### Frequency of vasculitis

The incidence of vasculitis in patients treated with ICIs is relatively low, estimated at around 1–2%. Risk factors for developing vasculitis include pre-existing autoimmune conditions, combination ICI therapy, and higher doses of ICIs [87]. Short-term outcomes can range from mild symptoms to severe organ damage, while long-term effects may include chronic vascular damage, increased risk of cardiovascular events, and potential recurrence of vasculitis.

## Screening for ICI-associated vasculitis

Screening for vasculitis involves several steps to accurately diagnose the condition and determine the affected vascular beds. The initial assessment includes a detailed medical history and physical examination to identify symptoms and potential risk factors. Blood tests identifying signs of inflammation, such as elevated C-reactive protein (CRP) and erythrocyte sedimentation rate (ESR), and specific antibodies like ANCA (anti-neutrophil cytoplasmic antibodies), which are associated with certain types of vasculitis [148] should be performed. Non-invasive imaging techniques, including ultrasound, Computed Tomography (CT), Magnetic Resonance Angiography (MRA), and Positron Emission Tomography (PET) scans, help visualize the affected blood vessels and organs, while angiography provides detailed images of blood vessels to detect abnormalities like aneurysms, a “string of beads” sign, or stenosis [147, 149, 150].

## Management of ICI-associated vasculitis

The guideline-based treatment of vasculitis induced by ICIs involves several key steps, primarily focusing on immunosuppression and supportive care. The first step is often to discontinue the ICI to halt the immune-mediated damage. High-dose corticosteroids are the mainstay of treatment, helping to reduce inflammation and immune activity, with typical regimens starting with high doses (e.g., prednisone 1–2 mg/kg/day) and gradually tapering based on clinical response. If corticosteroids are insufficient or if the patient relapses during tapering, additional immunosuppressive agents such as methotrexate, azathioprine, or mycophenolate mofetil may be used. In severe or refractory cases, biologic agents like rituximab (an anti-CD20 monoclonal antibody) or tocilizumab (an IL-6 receptor antagonist) should be considered. Supportive care includes managing symptoms and complications, such as thrombosis or organ ischemia, and providing supportive treatments like anticoagulation if needed [151–154].

## What are the unwanted effects of treating ICI cardiovascular toxicity?

High-dose steroids and immunosuppressive agents are commonly used in the management of ICI-induced cardiotoxicity as summarized above. However, these treatments come with potential unwanted effects that must be known to cardio-oncologists and include- hyperglycemia and fluid retention, infusion reactions, increased risk of infection, neutropenia and thrombocytopenia, transaminase elevation, and gastrointestinal perforation [155].

## Second line therapy for ICI toxicity and organ involvement

There is no single preferred immunosuppressant for ICI toxicity in general; the selection of immunosuppressants is often organ specific. As previously discussed, the ASCO guidelines recommend the use of corticosteroids as the first-line treatment for most irAEs. In steroid-refractory cases, additional immunosuppressive agents such as mycophenolate mofetil, infliximab, or abatacept may be used. The selection of additional immunosuppressants depends on the affected organ [45]:

- Gastrointestinal Toxicities: For moderate to severe colitis refractory to corticosteroids, infliximab or vedolizumab is recommended. Tofacitinib and ustekinumab are considered in refractory cases [156].
- Hepatotoxicity: For steroid-refractory hepatitis, mycophenolate mofetil is recommended. Infliximab is not recommended due to potential liver toxicity.
- Pneumonitis: Options include infliximab, mycophenolate mofetil, intravenous immune globulin (IVIG), or cyclophosphamide.
- Neuromuscular Toxicities: These are less common and may require specific immunosuppressants based on the clinical scenario (reviewed in Rossor) [157].
- Gastrointestinal: Vedolizumab, an anti-integrin α4β7 antibody, has shown promise in treating refractory colitis [158].
- Hepatitis: Mycophenolate mofetil is recommended for ICI hepatitis and nephritis.
- Cutaneous: Rituximab is often used for severe dermatologic irAEs like bullous pemphigoid [159].

Two ongoing randomized clinical trials—ACHLYS (NCT05195645) and ATRIUM (NCT05335928)—are investigating the role of abatacept in ICI-related myocarditis. Brief summaries are included below:

The **ACHLYS trial (NCT05195645)** is an ongoing phase II, randomized, double-blind, dose-finding study evaluating three intravenous abatacept dosing regimens (10, 20, or 25 mg/kg) in adult cancer patients with severe or corticosteroid-resistant ICI myocarditis. The trial randomizes approximately 21 patients (n = 7 per arm) and co-administers ruxolitinib and corticosteroids. The primary outcome is achieving ≥ 80% CD86 receptor occupancy on circulating monocytes after the first dose and sustained during the first 3 weeks. Secondary outcomes include immunological, myocardial, and muscular proxies of treatment response, as well as cancer progression-free and overall survival up to 1 year. The trial specifically addresses severe ICI myocarditis but also assesses the treatment’s impact on cardiac phenotypes such as arrhythmias, conduction abnormalities, and heart failure [160].

The **ATRIUM trial (NCT05335928)** is a phase III, multicenter, randomized, placebo-controlled study enrolling up to 390 hospitalized patients with ICI-associated myocarditis, as defined by the International Cardio-Oncology Society. Participants will receive either abatacept (10 mg/kg IV) or placebo at baseline, 24 h, and day 14, with an optional fourth dose at day 28, in addition to high-dose corticosteroids. The primary outcome is the incidence of major adverse cardiac events (MACE), a composite including cardiovascular death, non-fatal sudden cardiac arrest, cardiogenic shock, significant ventricular or bradyarrhythmias, or incident heart failure. Secondary outcomes include the individual MACE components, troponin levels, rates of VTE [161].

## Rechallenge in patients experiencing ICI toxicity

The American Society of Clinical Oncology (ASCO) provides specific guidelines for rechallenging ICIs in patients who have experienced ICI-induced toxicity. According to these guidelines, rechallenging ICIs can be considered when symptoms and/or laboratory values revert to ≤ grade 1 [45].

Key considerations include:

1. Severity of Initial Toxicity: For grade 3 toxicities, ICIs should be held, and high-dose corticosteroids (prednisone 1–2 mg/kg/day) should be initiated. If symptoms do not improve within 48–72 h, additional immunosuppressive agents like infliximab may be considered. Rechallenge is generally not recommended for grade 4 toxicities, except for endocrinopathies controlled by hormone replacement.
2. Patient-Specific Factors: The decision to rechallenge should consider the previous tumor response, duration of treatment, type and severity of the toxicity, time to toxicity resolution, availability of alternate therapies, and patient performance status (reviewed in Xu et al.) [162].
3. Risk of Recurrence: There is a notable risk of recurrence of the initial irAE upon rechallenge. For example, colitis, hepatitis, and pneumonitis have higher recurrence rates. A large pharmacovigilance cohort study reported a 28.8% recurrence rate of the initial irAE upon rechallenge.
4. Monitoring and Management: Close monitoring is essential during rechallenge. If rechallenged, patients should be monitored for early signs of recurrent toxicity, and prompt intervention should be available (Reviewed in Hue et al.) [163].

## The future of immune checkpoint inhibitors

Designing the next generation of ICIs to be safer and more durable involves several innovative strategies aimed at reducing irAEs while maintaining or enhancing therapeutic efficacy. Research is ongoing to develop new ICIs that target different immune checkpoints (LAG [Lymphocyte-activation gene]-3, TIM [T-cell immunoglobulin and mucin-domain containing]-3, and VISTA [V-domain Ig suppressor of T cell activation] [164, 165].

One promising approach is the use of functionally masked antibodies. These antibodies are conjugated with tumor microenvironment (TME)-responsive polymer chains, such as a mildly acidic pH-cleavable poly (ethylene glycol) (PEG) shell. This design minimizes systemic exposure and irAEs by preventing antibody-cell interactions at physiological pH, while restoring activity in the acidic TME, thus selectively targeting tumors [166].

Another strategy involves nanoparticle-mediated delivery systems. Stable nucleic acid lipid nanoparticles (SNALPs) can be used to deliver siRNA and mRNA constructs that simultaneously knock down inhibitory checkpoints (e.g., PD-L1) and induce stimulatory checkpoints (e.g., OX40L). This dual approach reprograms the TME from an immunosuppressive to an immunostimulatory phenotype, enhancing antitumor immunity with minimal toxicity [167].

Local and targeted delivery methods also show promise. For instance, transdermal microneedle patches and injectable hydrogels can provide sustained and localized release of ICIs, reducing systemic exposure and associated toxicities. These methods have demonstrated efficacy in preclinical models by facilitating the controlled release of antibodies at the tumor site [168].

Additionally, bispecific and tri-specific antibodies are being engineered to enhance selectivity and reduce off-target effects. These antibodies can simultaneously target multiple immune checkpoints or combine checkpoint inhibition with co-stimulatory signals, thereby improving therapeutic outcomes while minimizing adverse effects [169].

## Summary

ICIs, such as pembrolizumab, nivolumab, and atezolizumab, enhance immune responses against tumors by targeting PD-1, PD-L1, and CTLA-4 and offer an important option for treating cancer, particularly for advanced or metastatic disease. However, these treatments can also trigger severe cardiotoxic effects, including myocarditis, pericarditis, and myocardial infarction, with myocarditis being of particular concern. Other cardiac toxicities and vasculitis can occur during or after treatment. Effective screening and management of ICI-induced cardiotoxicity involves regular monitoring of cardiac biomarkers, electrocardiograms, and echocardiograms. When cardiotoxicity is suspected, immediate discontinuation of ICIs and administration of high-dose corticosteroids are recommended. In severe or steroid-refractory cases, additional immunosuppressive therapies, such as mycophenolate mofetil, infliximab, or abatacept are recommended as second-line therapy. A multidisciplinary approach involving oncologists and cardiologists is needed to optimize patient outcomes and manage ICI-induced cardiotoxicity effectively.

New targets, delivery systems, and tumor selectivity for ICIs promise to expand their reach and propel Immunotherapeutics well into the future [170].

## Data Availability

No datasets were generated or analysed during the current study.
