# Correction: Immune checkpoint inhibitors and cardiovascular toxicity: immunology, pathophysiology, diagnosis, and management

**DOI:** 10.1007/s11239-025-03234-8

**Published:** 2026-02-02

**Authors:** Richard C. Becker

**Affiliations:** Cardiovascular‑Oncology Program, University of Cincinnati Cancer Center, Cincinnati, OH 45267 USA

**Correction to: Journal of Thrombosis and Thrombolysis**

10.1007/s11239-025-03146-7

In this article, the legends for Figs. 1, 2, 3 and 4 were inadvertently omitted.

The figures should have appeared as shown below.

Figure 1:

**Fig. 1:**
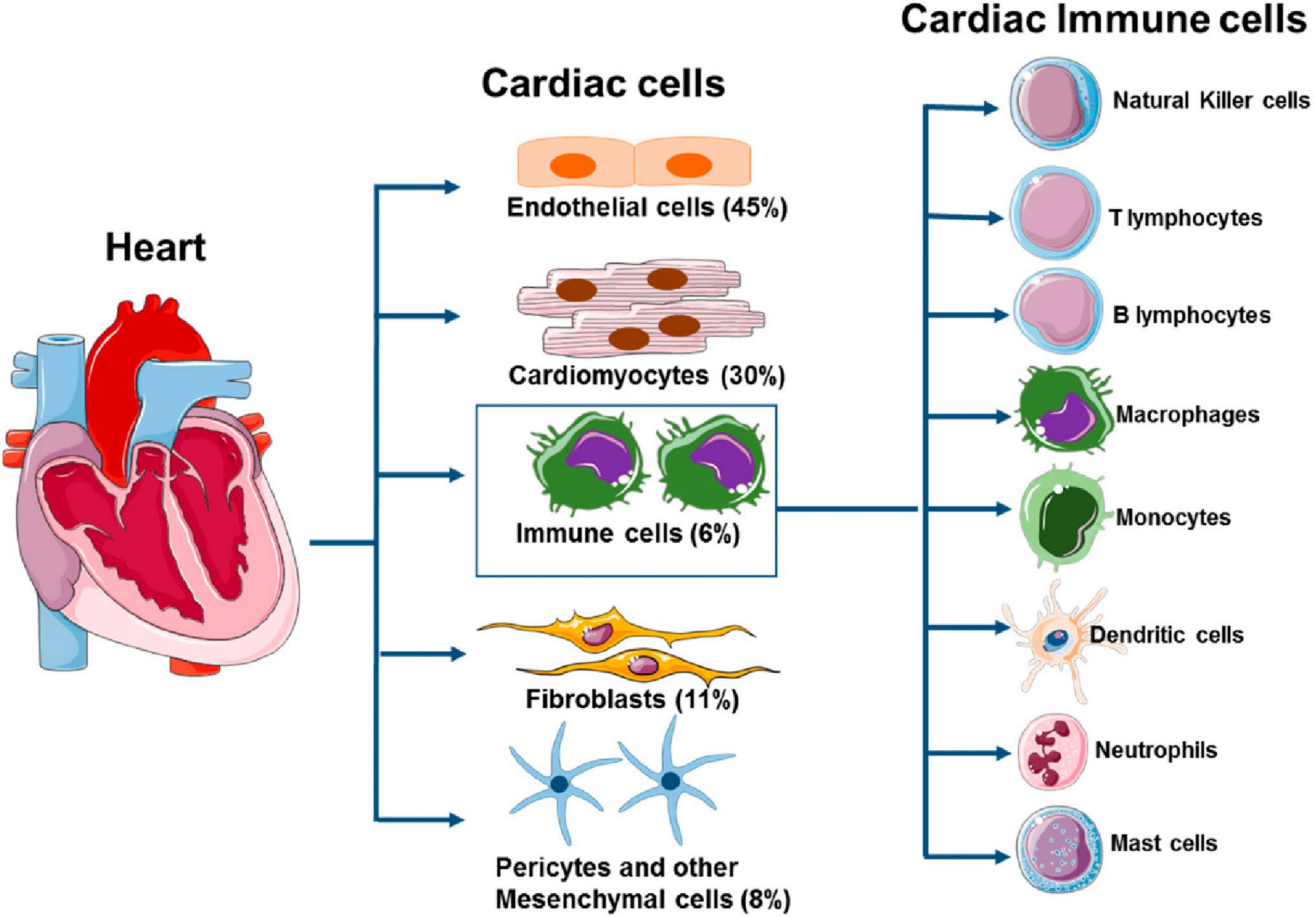
Immune System of the Heart. The heart has its own immune system, comprising various immune cells that play crucial roles in maintaining cardiac homeostasis and responding to injury. These immune cells are involved in essential housekeeping functions, such as removing dying tissue, scavenging pathogens, and promoting healing after myocardial infarction or infection. The primary immune cells include macrophages, dendritic cells, T cells, and B cells. Immune cells from the peripheral blood can also migrate to the heart when needed. The principal innate immune cells in the heart are *resident macrophages*, which perform phagocytosis of pathogens and apoptotic cells, regulate inflammation, and contribute to tissue repair. *Dendritic cells* are present in the myocardium and function as antigen-presenting cells, bridging innate and adaptive immunity by activating T cells. *Neutrophils* and *mast cells* are rapidly recruited during acute injury or infection, contributing to pathogen clearance and modulation of inflammation, but can also exacerbate tissue damage if uncontrolled. The adaptive immune system in the heart is primarily represented by *T lymphocytes* (including Th1, Th17, and regulatory T cells) and *B lymphocytes*. T cells orchestrate immune responses against infected or malignant cells, with regulatory T cells (Tregs) limiting excessive inflammation and promoting repair. B cells contribute to antibody-mediated immunity and modulate inflammation. *Natural killer (NK) cells* provide surveillance against virally infected and transformed (cancerous) cells, contributing to anti-tumor immunity within the cardiac microenvironment. See text. From: Lafuse, W.P.; Wozniak, D.J.; Rajaram, M.V.S. Role of Cardiac Macrophages on Cardiac Inflammation, Fibrosis and Tissue Repair. *Cells* 2021, *10*, 51. 10.3390/cells10010051

Figure 2:

**Fig. 2:**
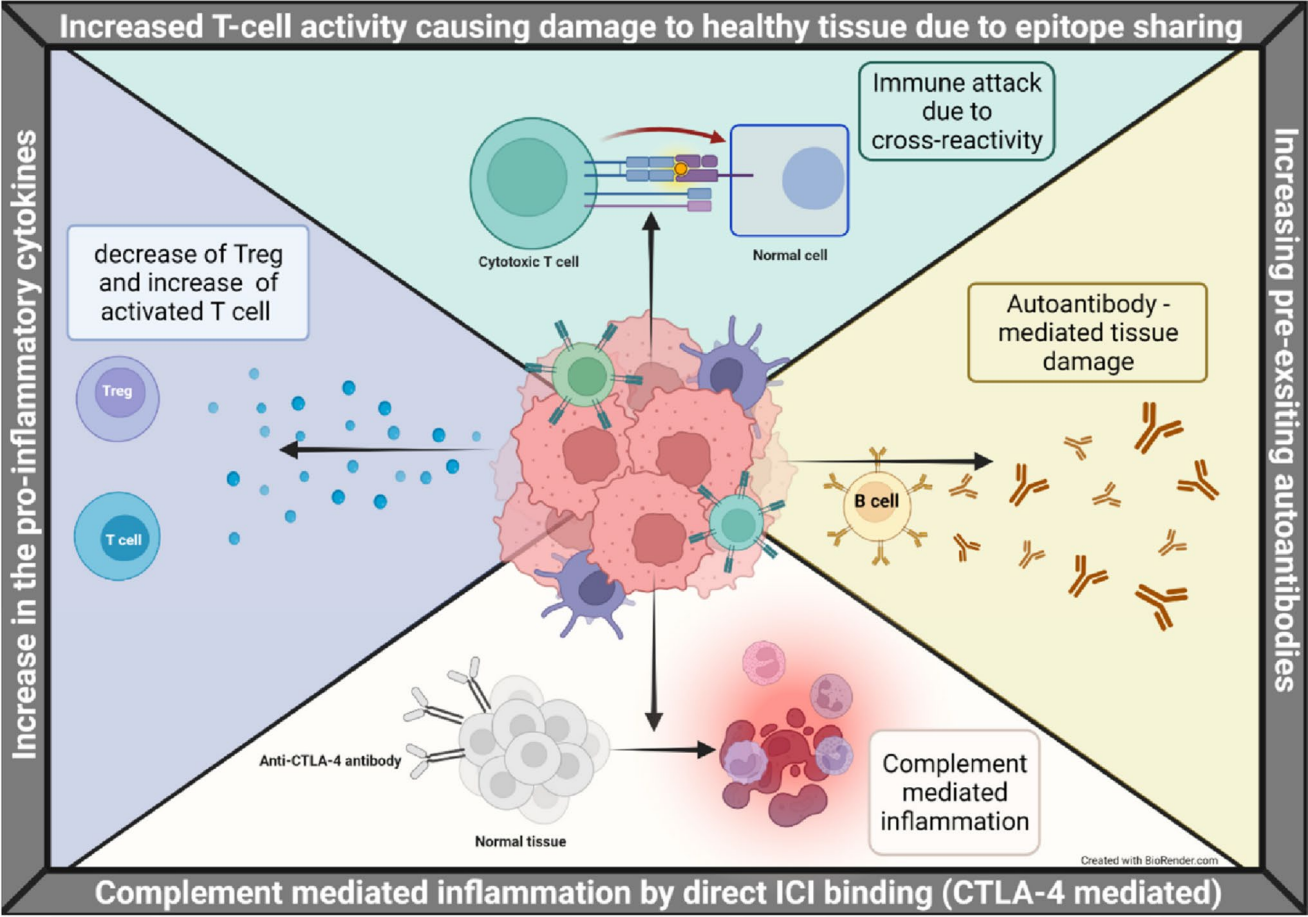
Proposed Mechanisms of Immune Checkpoint Inhibitor Toxicity. Immune checkpoint inhibitors (ICIs) and their respective ligands are shown in the context of the tumor immune microenvironment (TME). Various immune checkpoint target-mediated interactions between immune cells such as dendritic cells (DC) (serving as antigen presenting cells [APCs]), T cells, NK cells, and tumor cells are shown. ICIs disrupt immune homeostasis, leading to increased production of pro-inflammatory cytokines such as TNF-α, IL-1β, and IL-6, which can augment cardiac inflammation and dysfunction. Direct binding of antibodies against CTLA-4 with CTLA-4 expressed on normal tissues can enhance complement-mediated inflammation. From: Li, H.; Sahu, K.K.; Maughan, B.L. Mechanism and Management of Checkpoint Inhibitor-Related Toxicities in Genitourinary Cancers. Cancers 2022, 14, 2460. 10.3390/cancers14102460

Figure 3:

**Fig. 3:**
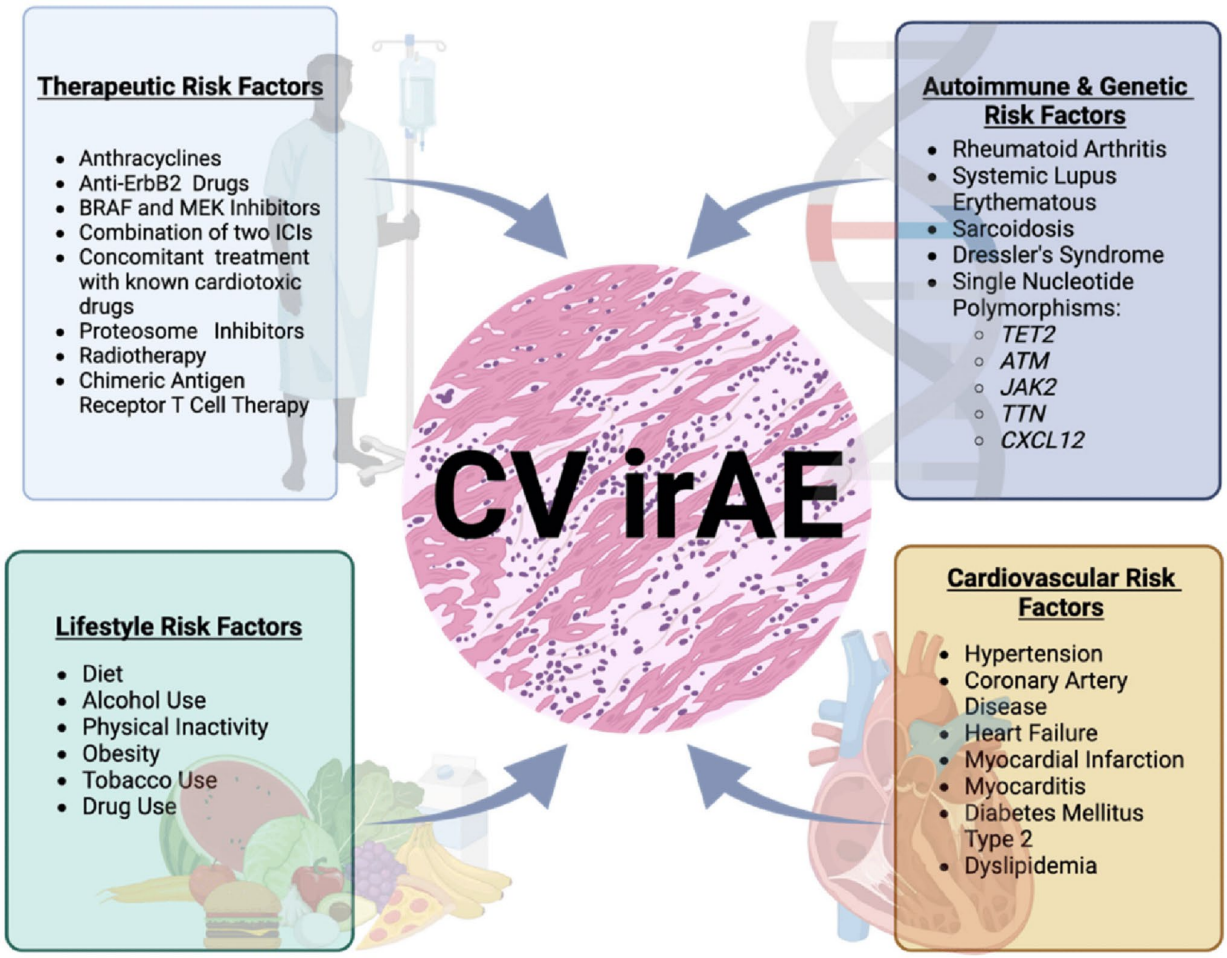
Patient-related Factors and ICI Cardiotoxicity. While the toxic effects of immune checkpoint inhibitors can be seen in a broad range of patients and cancers, there are patient-related factors that portend a higher risk. Combination therapy with immune checkpoint inhibitors (ICIs) and other agents—including BRAF/MEK inhibitors, tyrosine kinase inhibitors, radiation, and chimeric antigen receptor T-cell (CAR-T) therapy—has been associated with increased risk or potentially additive risk of cardiovascular immune-related adverse events, including myocarditis and other cardiotoxicities. The risk appears particularly elevated with ICI combination regimens and when ICIs are used alongside targeted therapies such as BRAF/MEK inhibitors or tyrosine kinase inhibitors, as these agents have independent cardiotoxic profiles and may have synergistic effects on cardiac risk. CAR-T cell therapy is also associated with cardiac toxicity, primarily in the context of cytokine release syndrome, and may further increase risk when combined with ICIs. Radiation, particularly thoracic radiotherapy, may have additive or synergistic effects with ICIs in increasing cardiac toxicity, but definitive data on the magnitude of this risk are still emerging. Preclinical and clinical data suggest overlapping mechanisms of injury, and combination regimens should be approached with caution. From: Green, C.E.; Chacon, J.; Godinich, B.M.; Hock, R.; Kiesewetter, M.; Raynor, M.; Marwaha, K.; Maharaj, S.; Holland, N. The Heart of the Matter: Immune Checkpoint Inhibitors and Immune-Related Adverse Events on the Cardiovascular System. *Cancers* 2023, *15*, 5707. 10.3390/cancers15245707

Figure 4:

**Fig. 4:**
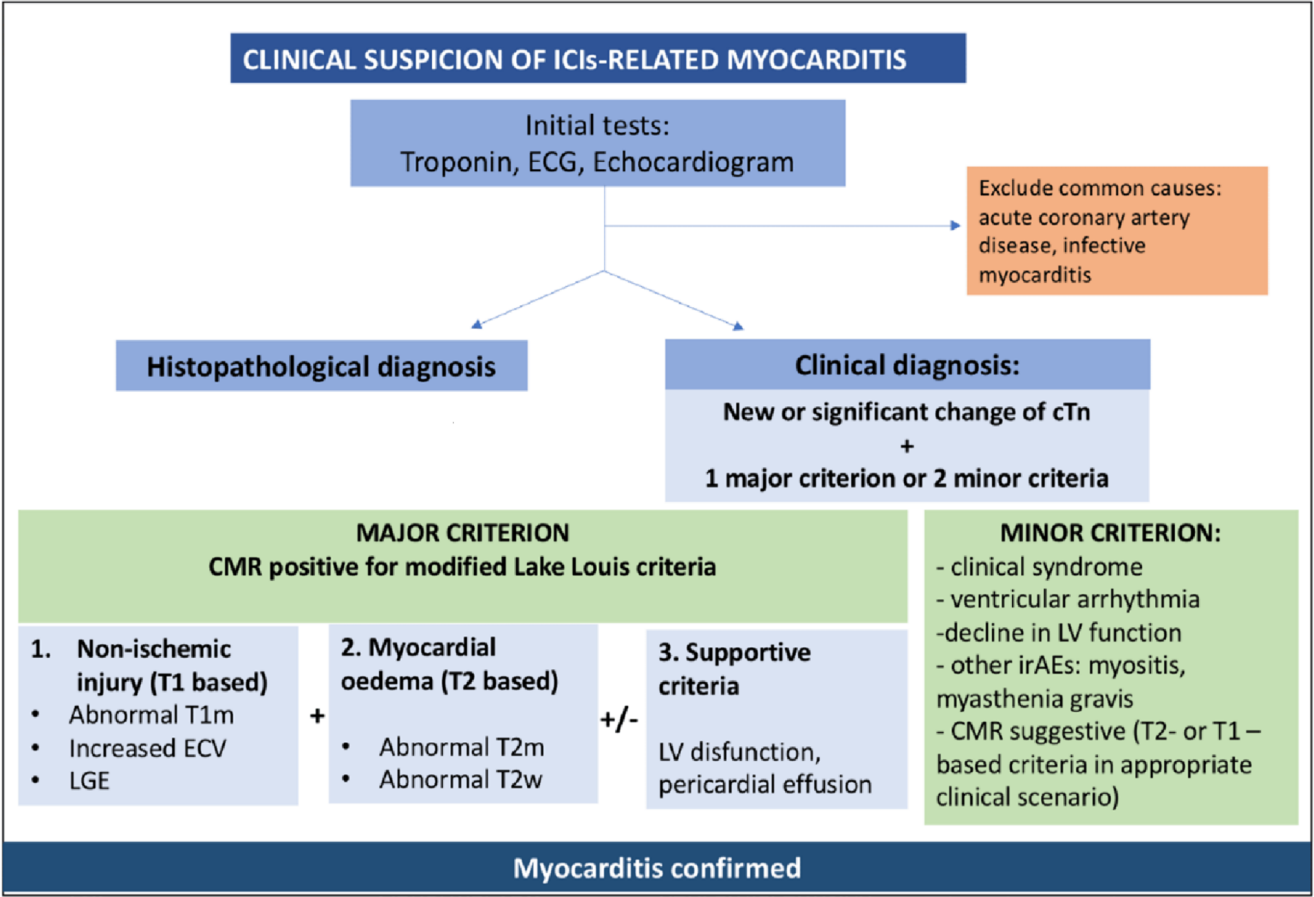
Screening Strategies to Detect ICI Cardiotoxicity. An early diagnosis of ICI-related cardiotoxicity is required for optimal management and outcomes. Baseline studies including an electrocardiogram, echocardiogram, brain natriuretic peptide (BNP) and high sensitivity troponin (hsTN) provide an important reference point or comparator for changes that might occur during treatment. Cardiac MRI is an important diagnostic modality. Major and minor criteria are important when the clinical suspicion is high. From: Frascaro, F.; Bianchi, N.; Sanguettoli, F.; Marchini, F.; Meossi, S.; Zanarelli, L.; Tonet, E.; Serenelli, M.; Guardigli, G.; Campo, G.; et al. Immune Checkpoint Inhibitors-Associated Myocarditis: Diagnosis, Treatment and Current Status on Rechallenge. *J. Clin. Med.* 2023, *12*, 7737. 10.3390/jcm12247737

The original article has been corrected.

